# RHAMM regulates MMTV-PyMT-induced lung metastasis by connecting STING-dependent DNA damage sensing to interferon/STAT1 pro-apoptosis signaling

**DOI:** 10.1186/s13058-023-01652-1

**Published:** 2023-06-22

**Authors:** Cornelia Tolg, Maja Milojevic, Freda W. Qi, Hailie A. Pavanel, M. Elizabeth O. Locke, Jenny Ma, Mathew Price, Andrew C. Nelson, James B. McCarthy, Kathleen A. Hill, Eva A. Turley

**Affiliations:** 1 London Regional Cancer Program, Lawson Health Research Institute, London, ON Canada; 2 Departments of Biology, Western University, London, ON Canada; 3 Departments of Computer Science, Western University, London, ON Canada; 4 Masonic Cancer Center, Laboratory Medicine and Pathology, University of Minnesota, Minneapolis, MN USA; 5 Departments of Biochemistry, Oncology and Surgery, Western University, London, ON Canada

**Keywords:** RHAMM, HMMR, Lung metastasis, STING, Interferon, apoptosis

## Abstract

**Background:**

RHAMM is a multifunctional protein that is upregulated in breast tumors, and the presence of strongly RHAMM^+ve^ cancer cell subsets associates with elevated risk of peripheral metastasis. Experimentally, RHAMM impacts cell cycle progression and cell migration. However, the RHAMM functions that contribute to breast cancer metastasis are poorly understood.

**Methods:**

We interrogated the metastatic functions of RHAMM using a loss-of-function approach by crossing the MMTV-PyMT mouse model of breast cancer susceptibility with *Rhamm*^*−/−*^ mice. In vitro analyses of known RHAMM functions were performed using primary tumor cell cultures and MMTV-PyMT cell lines. Somatic mutations were identified using a mouse genotyping array. RNA-seq was performed to identify transcriptome changes resulting from *Rhamm*-loss, and SiRNA and CRISPR/Cas9 gene editing was used to establish cause and effect of survival mechanisms in vitro.

**Results:**

*Rhamm*-loss does not alter initiation or growth of MMTV-PyMT-induced primary tumors but unexpectedly increases lung metastasis. Increased metastatic propensity with *Rhamm*-loss is not associated with obvious alterations in proliferation, epithelial plasticity, migration, invasion or genomic stability. SNV analyses identify positive selection of *Rhamm*^−/−^ primary tumor clones that are enriched in lung metastases. *Rhamm*^−/−^ tumor clones are characterized by an increased ability to survive with ROS-mediated DNA damage, which associates with blunted expression of interferon pathway and target genes, particularly those implicated in DNA damage-resistance. Mechanistic analyses show that ablating RHAMM expression in breast tumor cells by siRNA knockdown or CRISPR-Cas9 gene editing blunts interferon signaling activation by STING agonists and reduces STING agonist-induced apoptosis. The metastasis-specific effect of RHAMM expression-loss is linked to microenvironmental factors unique to tumor-bearing lung tissue, notably high ROS and TGFB levels. These factors promote STING-induced apoptosis of RHAMM^+ve^ tumor cells to a significantly greater extent than RHAMM^−ve^ comparators. As predicted by these results, colony size of Wildtype lung metastases is inversely related to RHAMM expression.

**Conclusion:**

RHAMM expression-loss blunts STING-IFN signaling, which offers growth advantages under specific microenvironmental conditions of lung tissue. These results provide mechanistic insight into factors controlling clonal survival/expansion of metastatic colonies and has translational potential for RHAMM expression as a marker of sensitivity to interferon therapy.

**Supplementary Information:**

The online version contains supplementary material available at 10.1186/s13058-023-01652-1.

## Introduction

Recent advances in early detection, and the development of targeted therapies have significantly improved clinical outcome of breast cancer patients [1–3], but metastasis and tumor recurrence remain as major obstacles to successful disease management [4]. Metastasis requires an ability of tumor cells to survive and expand in alien microenvironments and occurs as a result of a complex set of tumor and host traits that are distinct from those facilitating primary tumor initiation and growth. The metastatic process is still poorly understood at a mechanistic level [5, 6] although the discovery of promoter and suppressor genes that selectively affect metastases but not primary tumors has facilitated identifying tumor-intrinsic and microenvironmental properties necessary for secondary site colonization [6, 7].

One oncogenic signaling hub that has been implicated in the metastases of breast and other cancers is the multifunctional, intracellular/extracellular protein, RHAMM (gene name *HMMR*) [8–11]. RHAMM expression is heterogeneous in breast cancer, and the presence of strongly RHAMM-positive tumor cell subsets is linked to increased peripheral metastasis and poor clinical outcome [12]. Experimental models of breast cancer confirm a role for RHAMM in promoting functions associated with breast tumor initiation and metastasis [13–15] predicting that targeting this protein may improve clinical management of breast cancer metastases. Mechanistically, RHAMM performs multiple extracellular and intracellular functions relevant to metastasis [12, 16] including regulation of mitosis [17], genomic stability [18], cell motility, cellular plasticity [19–21], pluripotency of progenitor cells [20, 22] and oncogenic driver pathway activation [13]. In addition to tumor-intrinsic functions, RHAMM also regulates host cell responses that can impact tumor cell survival [8].

To better define the mechanisms by which RHAMM functions affect metastasis, we assessed the consequences of *Rhamm*-loss to mammary tumor progression using the MMTV-PyMT transgenic mouse model of breast cancer susceptibility [23]. This model was chosen for its rapid progression to metastatic disease, and its molecular similarity to both luminal B breast cancer and basal-like breast tumors, which typically express high levels of RHAMM clinically [24, 25]. *Rhamm*-loss in this model has no detectable effect on primary tumor initiation or growth but unexpectedly increases, rather than decreases, lung metastasis. This effect is traced to clonal selection of *Rhamm*^−/−^ tumor cells with an intrinsic resistance to DNA-damage-induced apoptosis that is sensed by STING/interferon signaling. This mechanism provides a survival advantage in lung but not the mammary microenvironment that is linked to the higher ROS and TGFB levels in lung tissue, which enhance STING-dependent apoptosis of RHAMM^+ve^ tumor cells but spare RHAMM^−ve^ comparators. These results identify RHAMM as a novel tissue-specific metastasis regulator and document tumor intrinsic and microenvironmental contexts that trigger its apparent metastasis suppressor functions.

## Material and methods

### Mouse breeding, genotyping, tumor measurements and whole mount preparations

C57Bl/6 *Rhamm*^−/−^ and Wildtype mice were crossed to MMTV-PyMT mice on an FVB background (purchased from Jackson Labs) as described by Lopez et al. [26] to obtain *Rhamm*^−/−^:MMTV-PyMT, *Rhamm*^±^:MMTV-PyMT and *Rhamm*^+/+^:MMTV-PyMT genotypes. The degree of SNP homozygosity, measured using the mouse diversity genotyping array (MDGA), was similar between *Rhamm*^−/−^:MMTV-PyMT and *Rhamm*^+/+^:MMTV-PyMT genotypes. Littermate heterozygotes (*Rhamm*^±^) from the *Rhamm*^±^:MMTV-PyMT x *Rhamm*^−/−^:MMTV-PyMT cross produced a similar tumor profile as the *Rhamm*^+/+^:MMTV-PyMT x C57Bl/6 Wildtype cross mice. The preparation and breeding of *Rhamm*^−/−^ mice, tumor measurements and whole mount preparations are described in Additional file 1: Methods.

Methods for IHC, IF, and immunoblot are described in [27] and in Additional file 1: Methods.

### CRISPR cell line generation

The MDA-MB-231 RHAMM CRISPR cell line was generated by transfection with paired guide RNA’s (5′–3′) GTATTGTATTTGATTAGAAT (within exon 3 of the *RHAMM* gene) and GAATTTGAGAATTCTAAGCT (within exon 6) in plasmid pCR4-TOPO-U6-HPRT-gRNA. Guide RNA’s were co-transfected with plasmid expressing the CAS9 enzyme (pT3.5 Caggs-FLAG-hCas9) as well as plasmids for puromycin and GFP selection, pcDNA-PB7 and pPB SB-CG-LUC-GFP (Puro)(+CRE), using Lipofectamine 2000 reagent (Invitrogen, cat#11668-019) following the manufacturer’s suggested protocol. Mock cell lines were generated by transfection of parent cells with selection plasmids only and selected as a pool by culture in puromycin containing medium (0.6 µg/ml). *RHAMM*-CRISPR knockout cell lines were selected by clonal plating in puromycin containing media (0.6 µg/ml). Single cell derived colonies were expanded and screened by genomic PCR for the corresponding deletion within the *RHAMM* gene (primers 5′–3′ AGATACTACCTTGCCTGCTTCA and ACCTGCAGCTTCATCTCCAT), and by immunoblot for loss of RHAMM protein.

### Primary cultures of tumor cells

Tumor cell isolation is described in Additional file 1:  Methods.

### Cultured cell treatment

For quantification of H_2_O_2_-induced apoptosis and STAT1 activation, *RHAMM*-CRISPR knockout and mock transfected cells were plated on cover slips in DMEM medium containing 10% FBS, resulting in sub-confluent cultures after 24 h incubation. STAT1 activation and apoptosis was induced by incubation in culture medium containing 50–200 µM H_2_O_2_ for either 4 (STAT1 activation) or 48–72 h (apoptosis). Cells were stained for either STAT1 or cleaved CASPASE 3 as described in Additional file 1: Methods. Staining quantification by ImageJ used confocal images.

To induce DNA double strand breaks or apoptosis in tumor cells that were isolated from primary tumors, cells were plated at low cell density on fibronectin coated coverslips and cultured in growth medium for 24–48 h. Cultures were treated with either Cis-platin or 300 µM H_2_O_2_ at the indicated concentrations and durations. Cells were stained for either γH2AX or ApopTag as described in Additional file 1:  Methods. Staining quantification by ImageJ used confocal images.

Py8119 cells were obtained from the ATCC (ATCC CRL-3278) and cultured in F12K medium (Wisent) supplemented with 5% FBS and Mito + Serum Extender. These cells were originally isolated and cloned from tumors that arose in C57Bl/6 MMTV-PyMT mice and therefore do not contain genomic sequences from FVB mice [28].

### siRNA transfection

Sub-confluent Py8119 tumor cells cultures were transfected using Lipofectamine RNAiMAX (Invitrogen) following the manufacturer’s instructions. Culture medium was changed to CTS Opti-MEM (Gibco). Lipofectamine RNAiMAX reagent was diluted 1:50 with CTS Opti-MEM. siRNA (ID: 151008, 159287, s279, negative control siRNA#1, Ambion) was diluted to a concentration of 500 nM in CTS Opti-MEM. Diluted transfection reagent and siRNA were mixed 1:1, incubated at RT for 20 min and then, added to the cell cultures. After 4–5 h incubation at 37 °C, medium was changed to culture medium containing STING agonists (Vadimezan (DMXAA), 33 µM, G10 for MDA-MB-231 tumor cells, 40 µM), H_2_O_2_ (300 µM), DMSO and/or TGFB1 (5 ng/ml). STAT1, CASPASE 3 staining or cell survival were analyzed 20–72 h later.

### AlamarBlue assay

Py8119 cells were plated at a density of 3000 cells/well of a 96 well plate using complete culture medium. After ON incubation at 37 °C, cells were transfected with *Stat1* siRNA, *Rhamm* siRNA or negative control siRNA. After transfection, cells were treated with F12K culture medium containing STING Agonist Vadimezan (33 µM), DMSO, H_2_O_2_ (300 µM), and/or TGFB1 (5 ng/ml). After 72 h incubation at 37 °C, the number of surviving cells was quantified by adding AlamarBlue reagent (1/10 Vol.) followed by 1–2 h incubation at 37 °C. Fluorescence was measured using a plate reader.

DNA and RNA isolation are described in Additional file 1:  Methods.

### Analysis of de novo mutation genotypes

The somatic (de novo) mutation burden in Wildtype and *Rhamm*^−/−^ MMTV-PyMT tumors were compared by identifying germline and mammary tumor mutations using a mouse genomic diversity array (MGDA) unbiased platform [29, 30]. The MDGA detects large, de novo postzygotic deletions and duplications as CNVs using close to 900,000 markers, and also de novo postzygotic base substitutions at single nucleotide polymorphic loci as SNVs, assayed at close to 500,000 SNV loci distributed across the mouse genome. Mouse diversity genotyping array (MDGA) hybridization was performed at the London Regional Genomics Centre (Robarts Research Institute, London, ON) according to instructions in the Affymetrix® Genome-Wide Human single nucleotide polymorphism (SNP) Nsp/Sty 6.0 manual (Affymetrix 2007; https://assests.thermofisher.com/TFS-Assets/LSG/manuals/snp6_atp_userguide.pdf). The resulting CEL files were then used for single nucleotide variant (SNV) genotyping and copy number variant (CNV) identification as described (Additional file 1: Methods). *Ilk*/*Rrp8, Taf10* genes (Mm00232271_cn), which overlap the same CNV region, were used for confirmation by ddPCR. The transferrin receptor gene (*Tfrc*) was used as the diploid copy number reference for the *Ilk*, *Taf10* and *Rhamm* assays. No-template controls and two technical replicates were used in all assays. DdPCR procedures are described in Additional file 1: Methods. Recurrent and unique CNVs were determined using HD-CNV (Additional file 1: Methods) [31]. To visualize CNV occurrence across the genome for individual mouse samples, a timeline-style plot was generated in R using ggplot2 (v3.2.1). Genic annotation used to identify the genic content of CNVs was obtained from Ensembl’s BioMart (Ensembl genes 67, NCBIM37). Protein-coding genes, non-coding genes, and pseudogenes that completely overlapped CNVs of the same state, in all three samples of a group (shared *Rhamm* genotype and tumor type), were considered recurrent within that group.

Phenogram construction was done as previously described [29] and is described in Additional file 1: Methods. Filtering procedures are described in Additional file 1: methods. Identification of candidate de novo mutations is described in Additional file 1: Methods. The spatial distribution of candidate de novo mutations across the genome was visualized using rainfall plots (Additional file 1: Methods) [32]. Chromosomes were treated linearly, and the genomic position of de novo mutations was assigned in an additive manner based on the position of the locus in relation to the whole GRCm38.p4 genome.

RNA sequencing is described in Additional file 1: Methods.

### Pathway analysis

Gene expression differences between *Rhamm*^*−/−*^ and Wildtype tumors of 1.5-fold with a p-value of less than 0.05 were analyzed for enriched Gene Ontology (GO) and KEGG pathways. The functions of differentially expressed genes were further probed using the hallmark gene set from the Molecular Signatures Database v 7.1 (https://www.gsea-msigdb.org/gsea/msigdb/index.jsp), used in the GSEA analysis, and Metascape (https://metascape.org/gp/index.html#/main/step1). Significantly down-regulated genes that grouped into the top hallmark gene sets in the GSEA analysis were assessed for mRNA co-expression (*p* < 0.05) with *RHAMM* (*HMMR*) using breast cancer data sets in cBioPortal (cbioportal.org). Invasive breast cancer subtypes that express the highest *RHAMM* mRNA levels were identified using the molecular subtypes in the TCGA PanCancer breast invasive carcinoma data set in cBioPortal (cbioportal.org).

Apoptag® staining, ROS/NOS and 8-oxodG ELISA are described in Additional file 1: Methods.

## Results

### *Rhamm*-loss increases metastasis in MMTV-PyMT mice but does not affect primary tumor initiation or growth

**Fig. 1:**
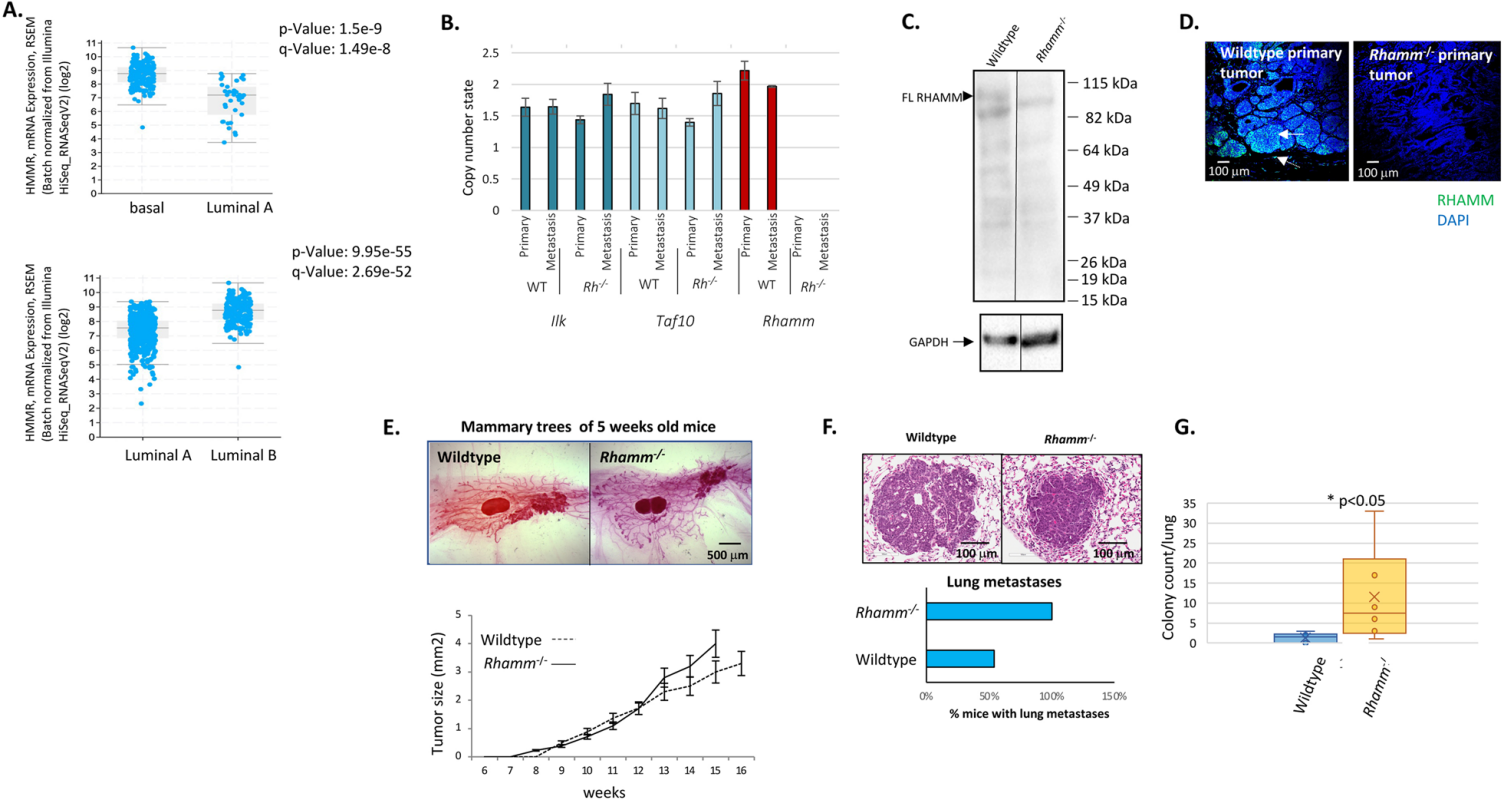
*Rhamm*-loss in MMTV-PyMT transgenic mice increases lung metastasis without affecting primary mammary tumor initiation, incidence or size. **A** Comparison of RHAMM mRNA expression in breast cancer subtypes (Breast Invasive Carcinoma, TCGA, PanCancer Atlas). RHAMM expression is significantly higher in basal and luminal B breast cancer subtypes than in luminal A comparators. **B** *Rhamm-*loss detected by the Mouse Diversity Genotyping Array (MDGA) genotyping was confirmed by ddPCR, which is the standard ultrasensitive method for verification of genotyping array-based CNV detection. Two additional ddPCR assays confirm another CNV deletion overlapping *Ilk* and *Taf10* genes that is detected by MDGA CNV genotyping, confirming accuracy of the MDGA and consistency of the two detection methods. WT = Wildtype; *Rh*^−/−^ = *Rhamm*^−/−^. **C** Western blot assays were performed using primary tumor lysates and RHAMM antibodies to the N-terminal sequence as described in Methods. Full-length RHAMM is expressed in Wildtype tumors (arrow) and is not detected in *Rhamm*^−/−^ tumors. **D** Histology sections of primary tumors from 16-week-old mice. Tissue was paraffin-processed, stained for RHAMM protein and imaged with a Nikon confocal microscope as described in Methods. Results show heterogeneous RHAMM staining in primary tumors and also in the host microenvironment (arrows). The lack of staining in histology sections from *Rhamm*^−/−^ tissue confirms the specificity of the anti-RHAMM antibody. **E** Whole mounts of mammary fat pads were prepared as described in Methods and show primary tumor initiation at 5 weeks in both Wildtype and *Rhamm*^−/−^ mice. Detectable primary tumor masses of Wildtype and *Rhamm*^−/−^ mice were measured weekly with calipers. Differences between genotypes are not statistically significant. Values are the Mean and S.E.M. *n* = 15 mice. **F** The lungs of Wildtype and *Rhamm*^−/−^ mice were harvested and metastatic nodules identified in hematoxylin/eosin-stained histology sections. 100% of *Rhamm*^−/−^ mice contain metastatic nodules in lungs in contrast to 58% of Wildtype mice, *n* = 11 mice/genotype. **G** Metastatic nodules were quantified from serial histology sections as described in Methods. *Rhamm*-loss significantly increases the number of metastatic colonies/lung compared to Wildtype. Values are the Mean and S.E.M. *n* = 15 mice. *p < 0.05

Data set analyses (cBioportal.org) confirm a previous report that RHAMM expression is highest in basal and luminal B breast cancer subtypes [33] (Fig. 1A, TCGA PanCancer Atlas, cBioportal). We utilized the MMTV-PyMT transgenic mouse, which models aggressive luminal B breast cancer [24, 34] to uncover oncogenic functions of RHAMM in an immune intact microenvironment, using loss-of-function, germline ablation of *Rhamm* [35].

For these experiments, we followed the breeding strategy of Lopez et al. [26] that resulted in *Cd44*^−/−^:MMTV-PyMT mice on a mixed C57Bl/6:FVB background. This study showed that *Cd44*-loss increased lung metastases, which was proposed to result from functional compensation by *Rhamm* [36]. We confirmed that *Cd44*-loss on this mixed background increases the incidence of lung metastasis (Additional file 2: Fig. S1). Successful deletion of *Rhamm* in *Rhamm*^−/−^:MMTV-PyMT mice was confirmed using the Mouse Diversity Genotyping Array (MDGA) and ddPCR (Fig. 1B), while absence of RHAMM protein expression was verified in *Rhamm*^−/−^ tumors by Western blot and immunohistochemistry (Fig. 1C, D). RHAMM full-length protein was confirmed to be expressed in Wildtype but not *Rhamm*^−/−^ MMTV-PyMT tumors (Fig. 1C), and immunofluorescent analyses further show that RHAMM protein expression is heterogeneous in Wildtype primary tumors, similar to human breast cancers [37], and also occurs in host cells of the tumor microenvironment (Fig. 1D).

As previously documented [38], Wildtype MMTV-PyMT primary tumors initiate in the mammary glands by 5 weeks (Fig. 1E) and reach a palpable size by 8 weeks (Fig. 1E, graph), while lung metastases are detected visibly as nodules and by histology at 14–16 weeks (Fig. 1F). *Rhamm*-loss does not detectably alter primary tumor initiation, incidence or growth (Fig. 1E) but results in increased lung colonies (Fig. 1F, G). Thus, 100% of *Rhamm*^−/−^ MMTV-PyMT mice develop lung tumors compared to 56% of Wildtype comparators (Fig. 1F), and metastatic colony number/lung is also significantly increased with *Rhamm*-loss (Fig. 1G). Dual loss of *Rhamm* and *Cd44* does not modify primary tumor initiation or growth, and these mice also exhibit elevated metastases similar to the single knockout of *Cd44* or *Rhamm* (Additional file 2: Fig. S1).

### Known RHAMM functions do not account for increased lung metastasis

RHAMM is well-documented to regulate a number of cellular processes in vitro that potentially affect metastasis including regulating tumor cell motility, invasion, cellular plasticity, proliferation and genomic instability [8, 15]. Analyses of primary *Rhamm*^−/−^ MMTV-PyMT tumor cell cultures show that *Rhamm*-loss significantly reduces both directed migration (scratch wound assays) and random motility speed (Timelapse assays) (Additional file 2: Fig. S2A, B). *Rhamm*-loss has no detectable effect on invasion, using standard Boyden chamber assays (Additional file 2: Fig. S2C), tumor cell proliferation (tumor size, Ki67 IHC, Additional file 2: Fig. S2D, E) or epithelial-to-mesenchymal (EMT) plasticity. Evidence for EMT was assessed by RNA-seq and immunofluorescence. RNA-seq analyses reveal no significant change in the expression of commonly used markers [39] for EMT (CDH1, CDH2, SNAI2, TWIST1, and FN1), while immunofluorescence analyses show no difference in protein staining for VIM and ZEB1 (Additional file 2: Fig. S2F).

**Fig. 2:**
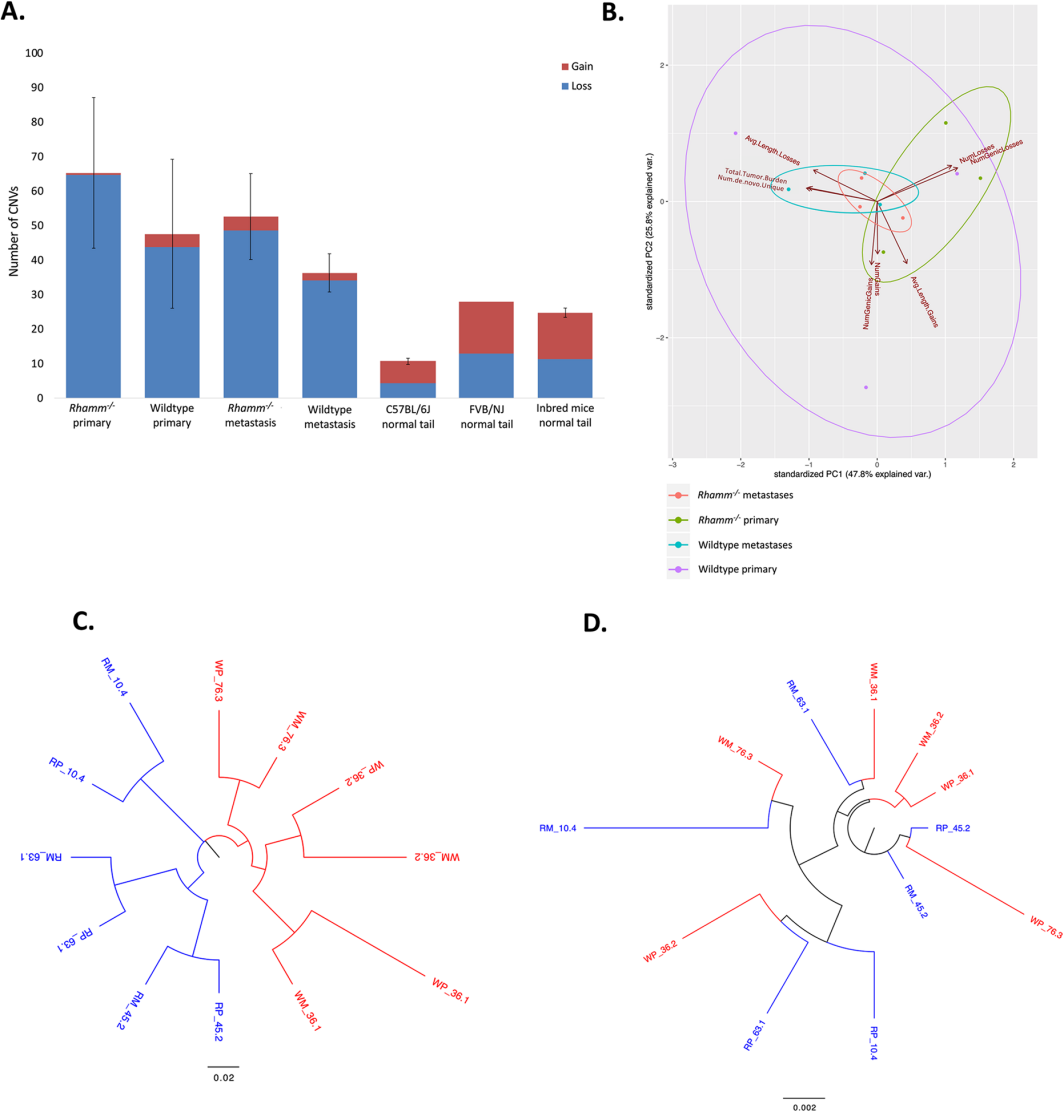

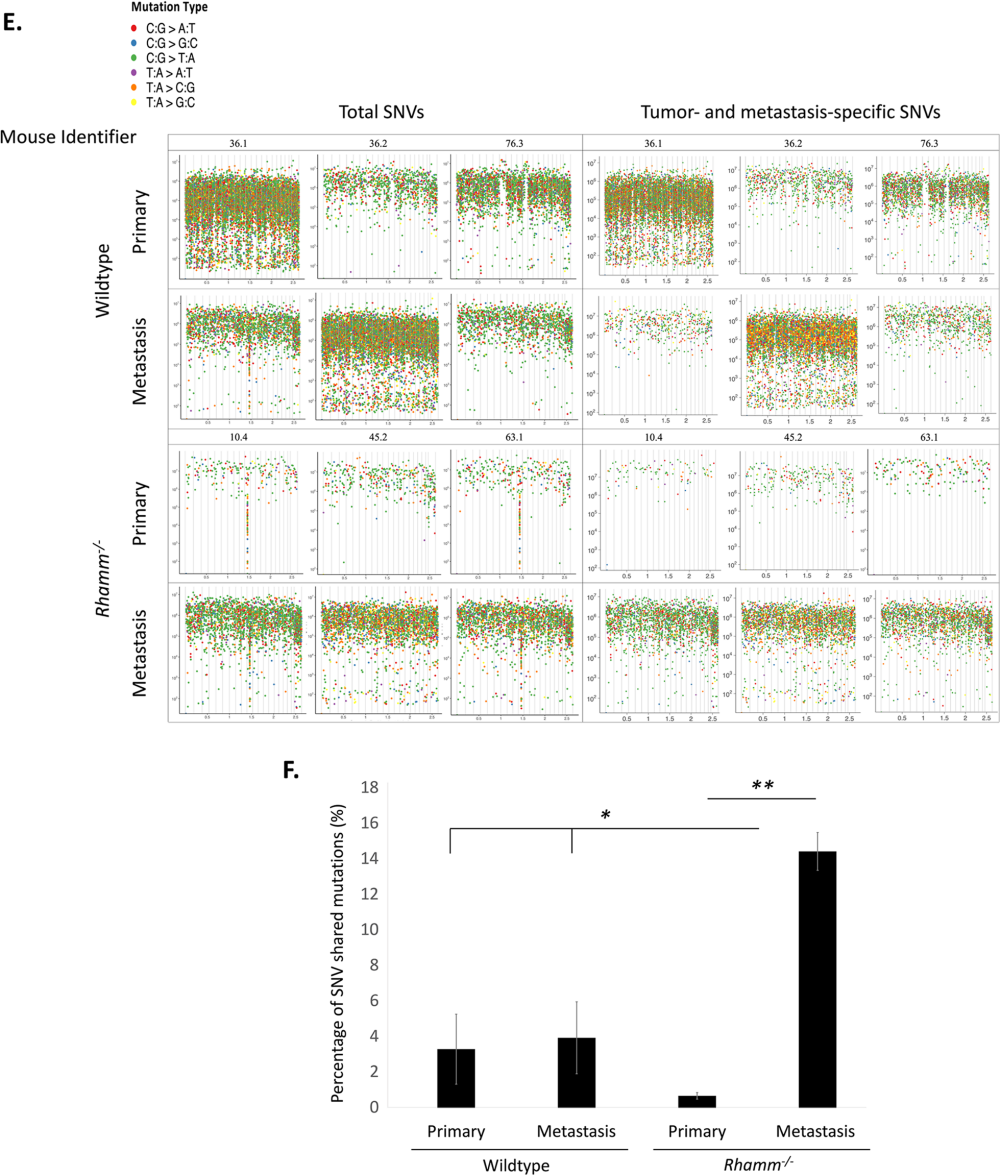
Wildtype and *Rhamm*^−/−^ tumor mutation burden is similar, but *Rhamm*^−/−^ tumors uniquely exhibit a strong inter-animal homogeneity in mutation characteristics. **A** The number of CNV gains and losses detected in primary tumor and metastases from Wildtype and *Rhamm*^*−/−*^ mice (*n* = 3/group) were compared with two inbred mouse stocks, C57Bl/6J (*n* = 8) and FVB/NJ (*n* = 1) and a reference set of inbred mice used for comparison with the inherited CNV burden (*n* = 114) as described in Methods. CNV gains are shown in red, while losses are shown in blue. Statistically significant differences between genotypes are not detected. Error bars represent standard error. B. Variation in the properties of de novo CNVs and SNVs in Wildtype metastases (WM), Wildtype primary (WP) tumors *Rhamm*^*−/−*^ metastases (RM) and *Rhamm*^*−/−*^ primary tumors (RP), (*n* = 3/group) is displayed as a principal component analysis (PCA). Results show clustering of these properties in *Rhamm*^−/−^ but not Wildtype tumors, indicating that the two genotypes differ in the CNV and SNV properties. **C**, **D** Phenograms representing SNV (**C**) and CNV (**D**) genetic differences. The numeric values in the sample labels represent the individual mouse identifier number. Scale bars represent genetic differences between samples. WP = Wildtype primary tumor; WM = Wildtype lung metastases; RP = *Rhamm*^−/−^ primary tumor; RM = *Rhamm*^−/−^ lung metastases. SNV and CNV differences were calculated as described in Methods. SNVs of *Rhamm*^−/−^ tumors clearly segregate from Wildtype comparators (**C**). Clear segregation of CNV differences between genotypes is not observed (**C**). **E** Different colors represent different mutation types: C:G > A:T (red), C:G > G:C (blue), C:G > T:A (green), T:A > A:T (purple), T:A > C:G (orange), T:A > G:C (yellow). *Rhamm*^−/−^ rainfall plots for both primary and metastatic tumors are similar between all three mice, while Wildtype rainfall plots vary, showing that a strong similarity in the pattern of SNV is acquired with *Rhamm*-loss. Results predict positive selection of a limited number of *Rhamm*^−/−^ tumor clones. F. SNV burden that is unique to Wildtype primary tumors, Wildtype lung metastases, *Rhamm*^−/−^ primary tumors and *Rhamm*^−/−^ lung metastases. There is a greater number of lung-specific SNVs in *Rhamm*^−/−^ metastasis vs. primary tumors, but these are unchanged in Wildtype tumors. Values are the Mean and S.E.M. *n* = 3 mice/group. **p < 0.01

**Fig. 3:**
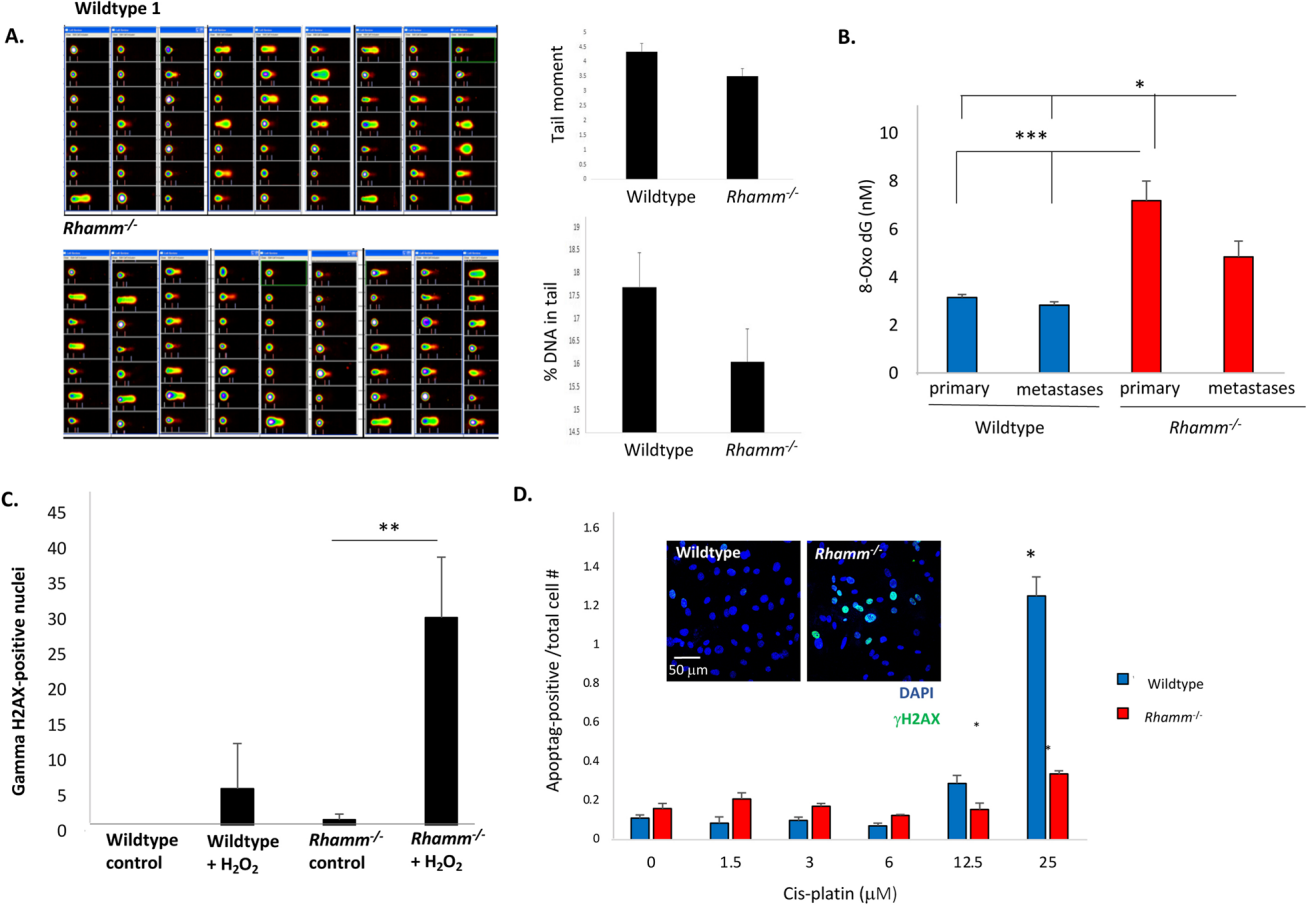
*Rhamm*^−/−^ tumor cells are resistant to DNA damage. **A** DNA fragmentation was quantified by comet assays as a measure of DNA damage using frozen samples of isolated, unstimulated Wildtype and *Rhamm*^−/−^ primary tumor cells. Quantification of % DNA in tail and tail moment is not affected by *Rhamm*-loss consistent with similar mutation burdens in the two genotypes identified by the genotyping array. Values are the Mean and S.E.M of *n* = 3 cell samples/genotype and *n* = 21 wells/genotype. **B** The amount of ROS-induced DNA damage in Wildtype and *Rhamm*^−/−^ tumors was quantified using ELISA as described in Methods. Results show that DNA from lung metastases of both genotypes carry more ROS-induced DNA damage that can cause point mutations and CNVs than primary tumors, which is consistent with the higher levels of ROS in lungs versus mammary fat pads. Values are the Mean and S.E.M. *N* = 4 biological replicates and 3 technical replicates. *p < 0.05, ***p < 0.001. C, D Cultures of Wildtype and *Rhamm*^−/−^ primary tumor cells were exposed to H_2_O_2_ (**C**) and cisplatin (**D**) to induce DNA damage. DNA damage was detected by gamma-H2AX staining, and cell death was quantified by Apoptag reactivity as described in Methods. *Rhamm*^−/−^ tumor cells survive with more DNA damage than Wildtype comparators. Values are the Mean and S.E.M. *n* = 3 biological replicates *p < 0.05, **p < 0.01

As expected, and independent of genotype, the total de novo mutation burden in MMTV-PyMT tumors is increased and CNVs are longer, have more copy number losses than gains, and occur more frequently within genes than the inherited germline CNV burden (Fig. 2A, Additional file 2: Tables S1, S2). The total mutation burden (Additional file 2: Table S1, S2, S3) and its genome-wide distribution in primary and metastatic tumors (Additional file 2: Fig. S3A) are not significantly (e.g., *p* > 0.05) altered by *Rhamm*-loss. There is also no obvious difference between genotypes in double- and single-strand DNA breaks as detected by Comet assays in Wildtype and *Rhamm*^−/−^ primary mammary tumor cells maintained in standard culture conditions (Fig. 3A) although CNV’s are uniquely shorter in *Rhamm*^−/−^ primary tumors (Additional file 2: Fig. S3B). Collectively, these results rule out obvious changes in motility, proliferation, plasticity and increased genomic instability associated with unrepaired DNA breaks and total mutation burden as mechanisms for the enhanced lung colonization observed in *Rhamm*^−/−^:MMTV-PyMT mice.

### *Rhamm*-loss alters the nature of the SNV mutation burden

A principal component analysis (PCA) of the collective de novo CNV and SNVs in Wildtype and *Rhamm*^−/−^ MMTV-PyMT primary and metastatic tumors was constructed to identify additional mutation patterns that might differ between the two genotypes (Fig. 2B) to provide clues for the increase in lung colonies resulting from *Rhamm*-loss. For CNVs, the number of gains, losses, average length of losses, average length of gains, number of losses overlapping or encompassing a gene and number of gains overlapping or encompassing a gene were assessed. SNV metrics included in the PCA are total mutations and number of de novo SNV genotype changes unique to each group (Additional file 2: Table S3). The PCA predicts that genetic variation in these collective properties is reduced by *Rhamm*-loss in both primary and metastatic tumors. A phenogram comparing genetic properties between tumors of the two genotypes was calculated using pairwise mutations at CNV and SNV loci. This shows that SNV properties clearly segregate *Rhamm*^−/−^ from Wildtype tumors (Fig. 2C) while, in contrast, CNV properties do not (Fig. 2D). These results predict that recurrent SNVs but not CNVs are associated with the metastatic progression as has previously been demonstrated in other pre-clinical models of breast cancer [40]. These results prompted us to further analyze the nature of SNVs in *Rhamm*^−/−^ vs. Wildtype tumors and their relationship to the increase in lung metastasis resulting from *Rhamm*-loss.

Graphic visualization of individual SNVs relative to the genome position (rainfall plots) provides an overview of mutation distribution and allows for pattern recognition (random vs. rainfall). Rainfall plots of SNVs show that the genome-wide distribution is similar in both genotypes (Fig. 2E), and that inter-tumor SNV heterogeneity is high in Wildtype tumors. However, SNV pattern heterogeneity is remarkably decreased in both the primary and metastatic tumors of *Rhamm*^*−/−*^ mice (Fig. 2E). Furthermore, while the total SNV burden and number of shared somatic SNV are similar in primary and lung Wildtype tumors, they are strongly increased by 100-fold in *Rhamm*^−/−^ lung vs. primary tumors (Fig. 2F, Additional file 2: Tables S1, S3). The majority of these shared de novo SNVs are non-genic (Additional file 2: Table S3).

These collective genetic analyses demonstrate that primary *Rhamm*^−/−^ and Wildtype primary tumors are significantly different from each other despite their similar growth and initiation properties in mammary tissue. The homogeneity of somatic SNV patterns predicts that total animal *Rhamm*-loss exerts positive or purifying selection for tumor cell clones within the mammary fat pad. We hypothesized that while these clones do not have a growth advantage in the mammary gland, their enrichment in lung metastases provide a selective advantage for survival in a lung microenvironment. Since the microenvironment of the lung is subject to greater oxidative stress than other tissues, the possibility that *Rhamm*^−/−^ tumor cells might be able to survive with more ROS-induced DNA damage than Wildtype comparators was next assessed.

### *Rhamm*-loss de-sensitizes tumor cells to DNA damage

Differences in oxidative stress of mammary vs. lung tissues were assessed by quantifying 8-Oxo-DG, used as a marker for oxidative damage (Fig. 3B). *Rhamm*^−/−^ primary tumors and lung metastases have significantly higher levels of 8-Oxo-DG than Wildtype comparators. While the comet assay did not reveal differences in single or double strand DNA breaks (Fig. 3A), the primary tumor cells used in this assay were not exposed to ROS-inducing agents. We therefore next compared the tolerance of primary *Rhamm*^−/−^ and Wildtype tumor cells to DNA damage in vitro by exposing primary cultures of tumor cells to ROS (H_2_O_2,_ Fig. 3C) or Cisplatin (Fig. 3D), both of which result in DNA damage and SNVs as well as CNVs. *Rhamm*^−/−^ tumor cells exposed to H_2_O_2_ or Cisplatin contain more DNA damage than Wildtype tumor cells, as detected by gamma H2AX staining. *Rhamm*^−/−^ tumor cells are also significantly more resistant to DNA-damage-induced apoptosis than Wildtype counterparts (Cisplatin shown, Fig. 3E), indicating that *Rhamm*-loss provides an intrinsic ability to survive DNA damage. The difference in this ability does not associate with altered expression of DNA repair enzymes, as detected by PCR arrays and RNA-seq, or with functions of shared de novo genic SNVs of *Rhamm*^−/−^ lung metastases predicted by IPA pathway analyses (Additional file 2: Fig. S4). Further analysis using RNA-seq was therefore performed to uncover mechanisms responsible for the increased ability of *Rhamm*^−/−^ tumor cells to survive with DNA damage.

### *Rhamm*-loss blunts interferon signaling

**Fig. 4:**
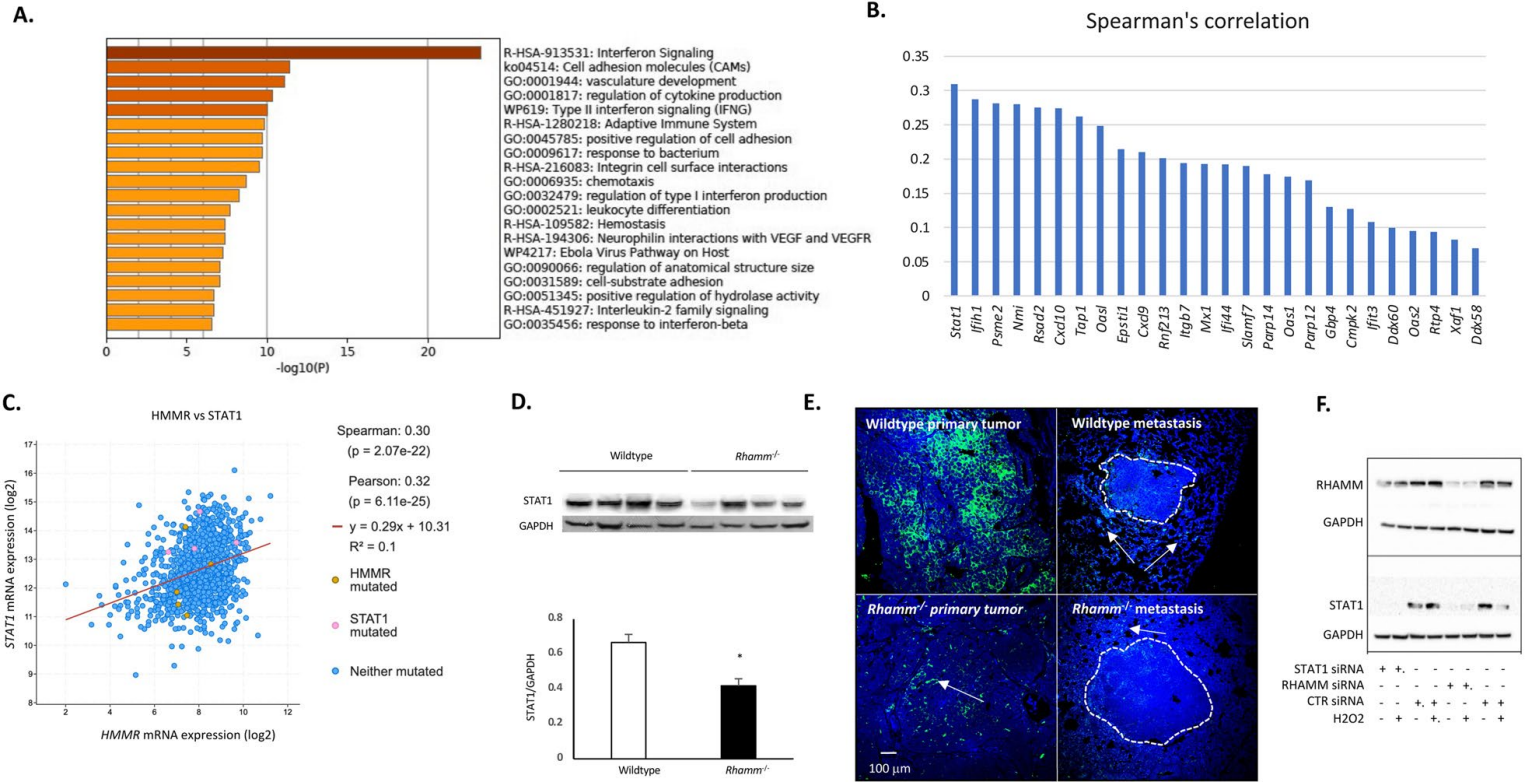
*Rhamm*-loss results in reduced expression of interferon alpha and gamma pathway components. **A** The transcriptome of primary MMTV-PyMT tumors was analyzed using RNA-seq as described in the Methods. Analysis of genes that are differentially expressed in Wildtype versus *Rhamm*^−/−^ primary tumors using Metascape and GSEA, identified interferon signaling as the top down-regulated pathway in *Rhamm*^−/−^ primary tumors. **B** GSEA Hallmark gene set analyses identified 51 IFNG and IFNA regulated genes as top hallmark gene sets that are down-regulated by *Rhamm*-loss. Twenty-six of these genes are significantly (*p* < 0.001) co-expressed with RHAMM in 2 datasets of breast cancer patients (METABRIC and TCGA Pan-Cancer Atlas, c-Bioportal.org) and are shown with Spearman correlation coefficients from METABRIC data sets in the histogram. **C** STAT1 is an example of an interferon pathway gene that is linearly co-expressed with RHAMM in luminal B breast cancer subtype. Graph is derived from the TCGA Pan-Cancer Atlas data set. **D** STAT1 protein levels in Wildtype and *Rhamm*^−/−^ primary mammary tumors were analyzed by Western blot. Wildtype tumors express significantly more STAT1 protein compared to *Rhamm*^−/−^ tumors. Values are the Mean and SEM of *n* = 3 tumors. *p < 0.05. **E** STAT1 staining of Wildtype and *Rhamm*^*−/−*^ primary tumors and lung metastases. Wildtype tumors contain more STAT1-positive cells in tumors and the tumor microenvironment than *Rhamm*^−/−^ counterparts. **F** *Rhamm* mRNA expression was knocked down by siRNA in the Py8119 MMTV-PyMT mammary tumor cell line as described in Methods, and the effect on RHAMM and STAT1 protein expression was quantified using Western blots. *Rhamm* knock-down strongly reduces STAT1 protein expression

RNA-seq shows that *Rhamm*-loss decreases expression of 459 genes and increases expression of 168 genes in primary mammary tumors (*p* < 0.05). Unbiased pathway analyses of significantly (*p* < 0.05) upregulated genes using the Molecular Signatures Database (v7.1) and Metascape reveal a weak association with aberrant G2M checkpoint (3.03e−4), E2F (1.69e−3), MYC signaling (1.06e−2) and cell cycle (Additional file 2: Fig. S5B), respectively. Metascape analyses of down-regulated genes predict strongly suppressed interferon signaling (e.g., Fig. 4A), and hallmark gene set analyses (GSEA) reveal significant down-regulation of gene sets in *Rhamm*^−/−^ tumor cells that respond to inflammatory stimuli (Additional file 2: Table S4). The top gene sets are regulated by IFNG (*p* = 2.43e−61) and IFNA (*p* = 4.54e−39) (Additional file 2: Table S4). Expression of IFNG and IFNA cytokines are not downregulated, but the expression of 53 (IFNG) and 31 (IFNA) pathway and target genes are significantly reduced (approximately twofold) by *Rhamm*-loss (*p* < 0.05). Twenty-two of these genes are coordinately expressed with RHAMM mRNA in 2 breast cancer data sets (METABRIC, TCGA Cancer Atlas cBioportal, Fig. 4B). Notably the breast cancer tumor suppressor and interferon-regulated transcription factor STAT1 [41] is most strongly co-expressed with RHAMM in these data sets (Fig. 4B, C, TCGA pan-cancer atlas shown). STAT1 protein levels are significantly reduced in *Rhamm*^−/−^ MMTV-PyMT primary tumors as detected by Western blots (Fig. 4D–F). Immunofluorescence assays show that STAT1 protein is reduced in both tumor and host cells of *Rhamm*^−/−^ primary tumors and lung metastases (Fig. 4E). Silencing RHAMM expression by siRNA knockdown in Py8119 MMTV-PyMT tumor cells also reduces STAT1 protein expression (Western blot, Fig. 4F).

### *Rhamm*-loss impairs STING-IFN signaling that senses DNA damage

IFN signaling is complexly associated with experimental and clinical tumorigenesis [42]. Robust IFN signaling is cytotoxic and linked to improved outcome in both luminal B and TNBC/basal-like breast cancer molecular subtypes [43, 44]. However, chronic, low activation of IFN signaling provides pro-survival advantages to cancer cells [45]. Notably, a subset of IFN-regulated genes that constitute an IFN-related DNA damage signature (IRDS), which includes STAT1[46], provides protection against DNA damage sensed by cGAS-STING in breast cancer cell lines [47]. Eighteen of these IRDS genes are regulated by RHAMM expression and are co-expressed with RHAMM in breast cancer datasets (Additional file 2: Table S5). Since blocking IFN signaling increases metastasis but not primary tumor growth in MMTV-PyMT mice [48] and forced RHAMM expression activates cGAS-STING in a BRCA1 mouse model of breast cancer susceptibility [14], we assessed if *Rhamm*^−/−^ cells have an aberrant cGAS-STING-IFN signaling, which provides a survival advantage in lung tissue. The effects of *Rhamm*-loss on STING-activated interferon signaling and tumor cell survival were further investigated using MMTV-PyMT tumor cells and the human basal-like MDA-MB-231 breast tumor cell line.

**Fig. 5:**
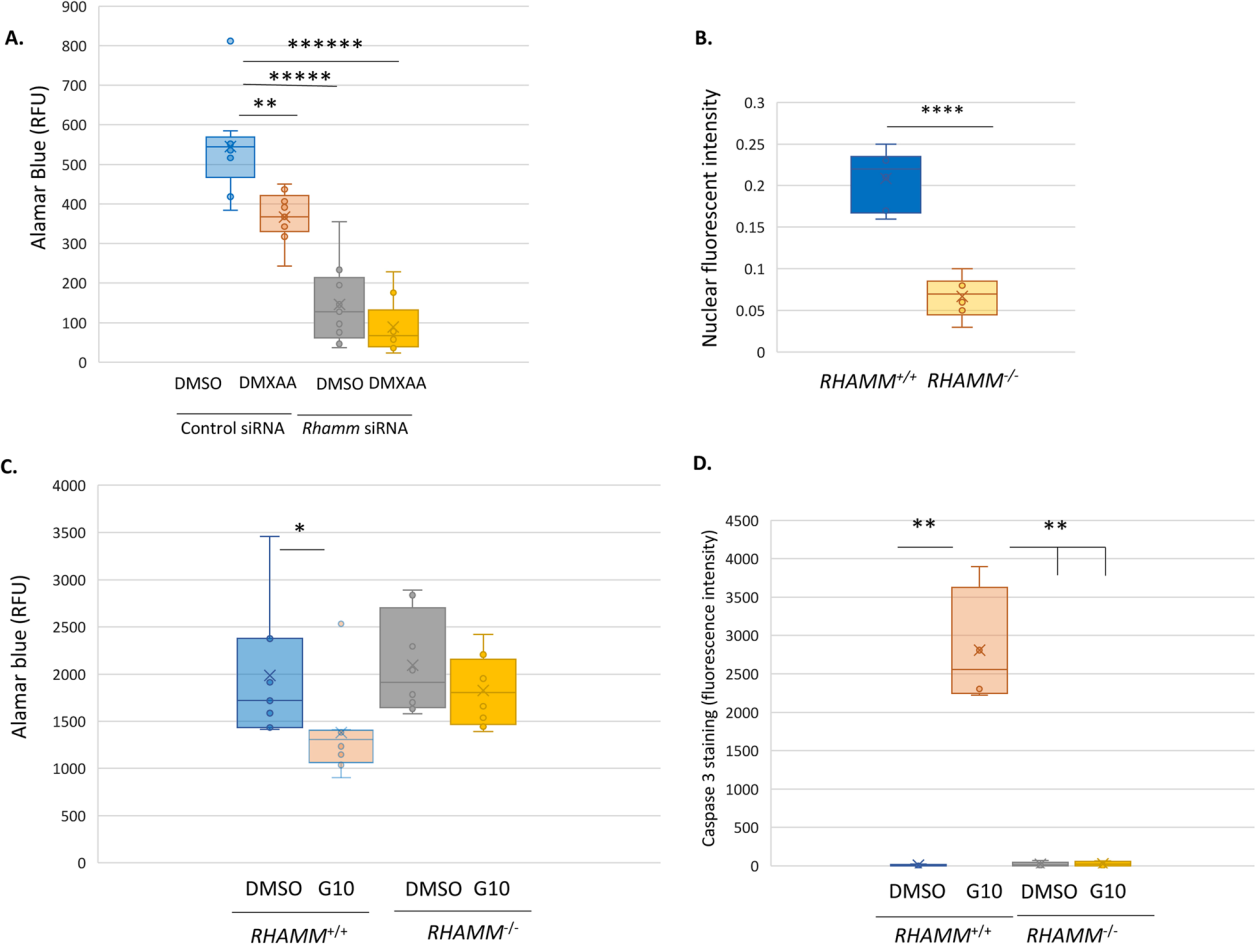
*Rhamm*-loss blunts response to STING-activated interferon signaling. **A** Py1189 MMTV-PyMT tumor cells were exposed to the mouse STING agonist, MDXAA, and the effect on tumor cell survival was quantified with AlamarBlue as described in Methods. MDXAA reduces the survival of *Rhamm*^+/+^ Py1189 tumor cells but has no significant effect when *Rhamm* is knocked down. Values are the Median and SD, *n* = 8 replicates. **p < 0.01, ****p < 0.00001, ******p < 0.000001. **B** STAT1 activity in the STING agonist, G10-stimulated *RHAMM*^+/+^ and *RHAMM*^−/−^ MDA-MB-231 tumor cells were measured by quantifying nuclear STAT1 protein as described in Methods. Results show that *RHAMM*-loss significantly reduces STAT1 activity. Values are the median and SD. *n* = 30 cells/genotype. ****p < 0.0001. **C**, **D** *RHAMM*^+/+^ and *RHAMM*^−/−^ MDA-MB-231 tumor cells were exposed to the human STING agonist G10, and survival was quantified using AlamarBlue (**C**) and cleaved Casapse3 (**D**) as described in Methods. Results show that similar to the MMTV-PyMT tumor cells, *RHAMM*-loss in MDA-MB-231 cells generates resistance to G10-induced cell death. Values are the Median and Std. Dev. *n* = 6 replicates. *p < 0.05, **p < 0.01

The consequence of RHAMM knockdown by siRNA to STING-mediated MMTV-PyMT tumor cell line survival was assessed using the STING agonist DMXAA. DMXAA significantly reduces RHAMM^+ve^ tumor cell survival but not RHAMM^−ve^ tumor cells (Fig. 5A). Deletion of genomic RHAMM in the human basal-like MDA-MB-231 breast tumor cell line ablates RHAMM protein expression (Tarullo SE, 2023. J. Pathol. 2023 Jul;260(3):289–303) and significantly reduces activation of STAT1 by the STING agonist, G10, as detected by decreased STAT1 nuclear localization relative to *RHAMM*^+*/*+^ mock-transfected cells (Fig. 5B). Further, G10 significantly decreases survival of *RHAMM*^+/+^ but not *RHAMM*^−/−^ MDA-MB-231 cells as detected by AlamarBlue (Fig. 5C) and cleaved CASPASE3 (Fig. 5D). These results identify an impaired response to STING-induced cell death as an intrinsic property of *Rhamm*^−ve^ mammary tumor cells.

### The ***Rhamm***^−/−^ lung microenvironment contains high levels of ROS and TGFB

**Fig. 6:**
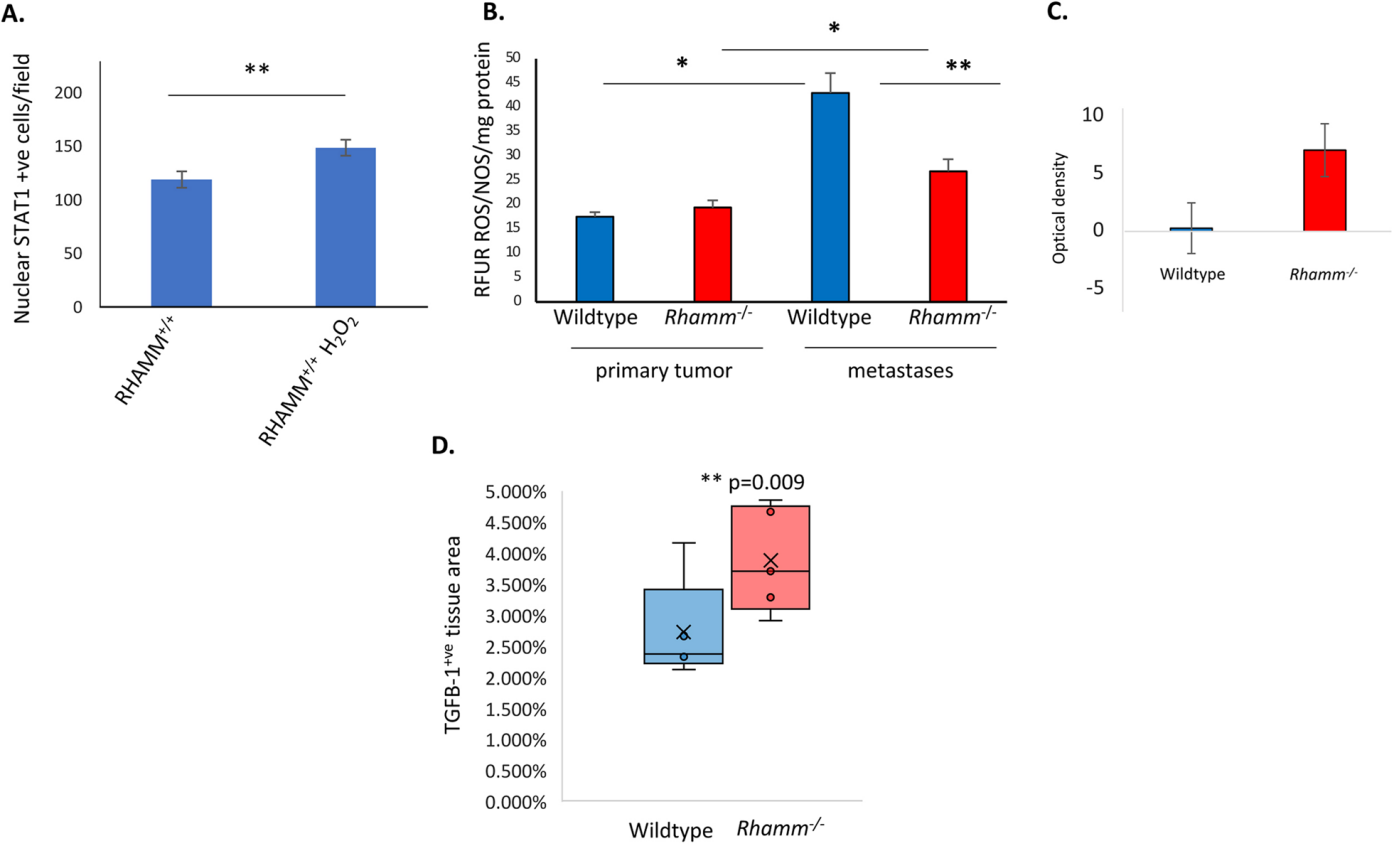
The tumor microenvironments of lung and mammary fat pads differ, but are minimally altered by *Rhamm*-loss. **A** MDA-MB-231 *RHAMM*^+/+^ tumor cells were exposed to H_2_O_2_, and the effect on STAT1 activation was quantified as the number of cells/field with nuclear STAT1 immunofluorescence. H_2_O_2_ significantly increases STAT1 activation. Values are the Mean and S.E.M. *n* = 5 fields. **B** Reactive oxygen species (ROS/NOS) levels in Wildtype and *Rhamm*^−/−^ primary and metastatic tumors were quantified using ELISA as described in Methods. Results show that levels are similar in tumors of both genotypes. Notably ROS levels are higher in lung vs. primary tumors from both genotypes. Values are the Mean and S.E.M. of *n* = 4 biological replicates and 3 technical replicates *p < 0.05, **p < 0.01. **C** IFNG protein levels were measured by antibody array in Wildtype and *Rhamm*^−/−^ lungs with metastases as described in Methods. IFNG protein is highly variable in both genotypes but not significantly different. Values are the median and Std. Dev. *n* = 3 replicates. **D** TGFB protein was quantified by IHC in primary and lung tumors. TGFB levels are significantly higher in *Rhamm*^*−/−*^ lungs than in Wildtype counterparts. Values are the Mean and S.E.M. of *n* = 5 lungs/genotype

We next compared lung and mammary fat pad microenvironments to identify properties that might provide a selective survival advantage for STING-resistant *Rhamm*^−/−^ tumor cells in lung tissue. ROS/NOS exposure results in DNA damage, stimulation of cGAS-STING, which senses this damage, and a consequent promotion of IFN-mediated cell apoptosis [42, 49]. We therefore confirmed that exposure to H_2_O_2_ at levels that damage DNA (e.g., Fig. 3D), activate interferon signaling as detected by increasing STAT1 nuclear localization in *RHAMM*^+/+^ tumor cells (Fig. 6A). ROS/NOS levels (Fig. 6B) were next shown to be significantly higher in lung than in mammary tissue of both genotypes (Fig. 6B). Consistent with RNA-seq data, IFN protein levels are collectively similar in the primary and metastatic tumors of both *Rhamm*^−/−^ and *Rhamm*^+/+^ (Wildtype) genotypes (Fig. 6C, primary tumors shown). Since elevated ROS levels are not specific to the *Rhamm*^−/−^ genotype, we examined other properties of the lung microenvironment that might contribute to the enhanced metastasis observed in *Rhamm*^−/−^ lungs. We compared the protein levels of TGF-beta1 in Wildtype and *Rhamm*^−/−^ tumor-bearing lungs since this cytokine is a metastasis promoter in breast cancer [50, 51] that is linked to RHAMM functions, DNA damage, and regulation of interferon signaling [52]. TGFB mRNA expression does not differ in primary *Rhamm*^−/−^ vs. Wildtype tumors as detected by RNA-seq but protein levels are significantly increased in tumor-bearing *Rhamm*^−/−^ lungs relative to Wildtype counterparts (Fig. 6D). Collectively, these results reveal ROS levels as a key difference in lung vs. mammary fat pad microenvironments that likely affect lung colonization and further identify an elevated expression of the immune-suppressive TGFB cytokine specifically detected in *Rhamm*^*−/−*^ lung tissue that might favor increased growth and survival of tumor cells with RHAMM expression-loss.

### ***Rhamm***^−/−^ tumor cells are resistant to ROS and TGFB-induced cell death

**Fig. 7:**
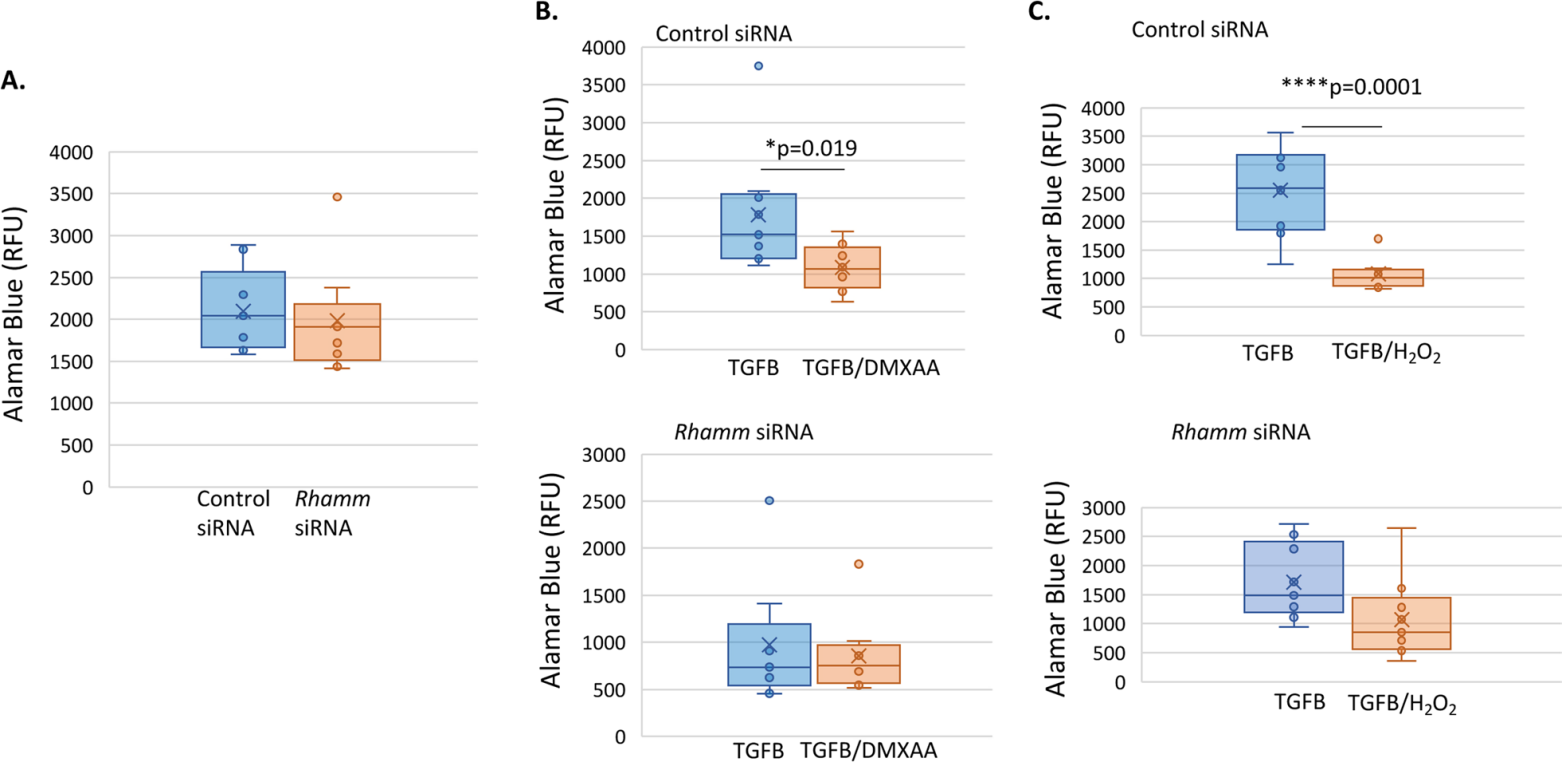
*Rhamm*-loss alters the response of tumor cells to the lung microenvironment. **A**–**C** The PyMT 8119 cell line was exposed to culture medium alone, TGFB alone or TGFB together with DMXAA or H_2_O_2_ then cell survival quantified as described in Methods. **A** Survival of *RHAMM*^+ve^ and *RHAMM*^−ve^ tumor cells is similar in culture medium alone. **B**, **C** The addition of TGFB with (**B**) DMXAA or (**C**) H_2_O_2_ reduce RHAMM^+ve^ but not RHAMM^−ve^ tumor cell survival. The line indicates the Mean and S.E.M. of *n* = 8 samples. *p < 0.05, ****p < 0.0001

We simulated a ROS/TGFB-high lung microenvironment by exposing RHAMM^+ve^ and RHAMM^−ve^ MMTV-PyMT tumor cells to combinations of ROS, TGFB and STING agonists then quantified the consequence to tumor cell survival. The survival of RHAMM^+ve^ and RHAMM^−ve^ tumor cells is not differentially affected in standard culture conditions of low ROS and TGFB levels (Fig. 7A). In contrast, exposure to TGFB combined with either H_2_O_2_ or STING agonist DMXAA significantly decreases the survival of RHAMM^+ve^ but not RHAMM^−ve^ tumor cells (Fig. 7B, C). Thus, tumor cells with RHAMM expression-loss are impaired in their ability to sense DNA damage, which is associated with an impaired response to STING-agonists and with a down-regulation of IFN pathway and target genes. This aberrant response provides a survival advantage in a high ROS and TGFB microenvironment.

**Fig. 8:**
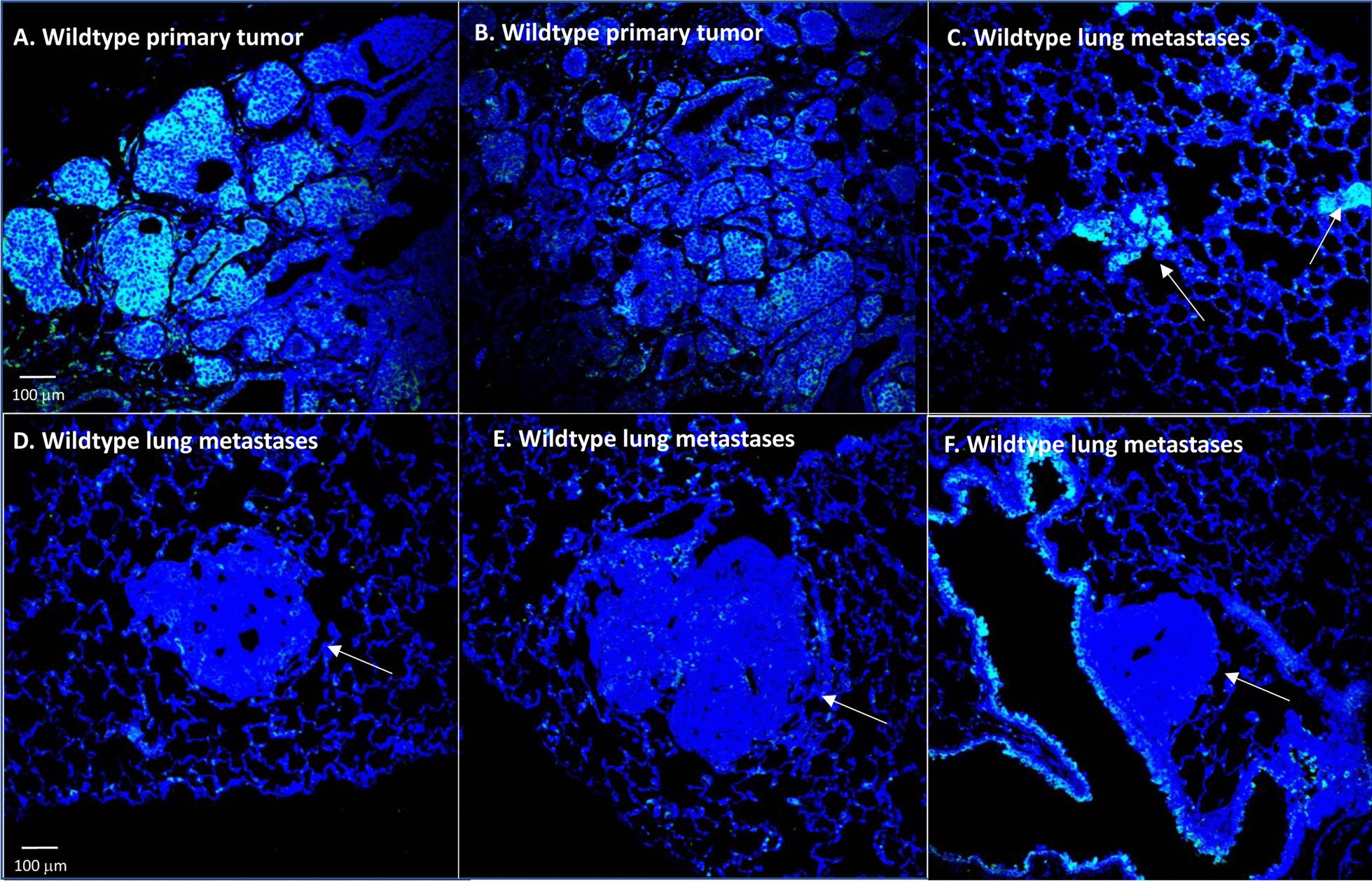
RHAMM expression is heterogeneous in wildtype primary tumors of MMTV-PyMT mice but is reduced in lung metastases. **A**, **B** Wildtype primary MMTV-PyMT tumors express RHAMM in a heterogeneous manner. **C**–**F** show the varying levels of RHAMM expression in lung colonies of wildtype mice. RHAMM expression is highest in small lung colonies (**C**), and low (**D**) to negative (**E**, **F**) in larger colonies. RHAMM is consistently expressed in the lung microenvironment (**C**–**F**)

These collective results predict that survival of wildtype lung colonies would also benefit by low or absent *Rhamm* expression, although not to the extent of a *Rhamm*^−/−^ lung microenvironment. To assess this possibility, tumor-bearing, Wildtype lung tissue sections were stained for RHAMM and STAT1. As shown in Fig. 8, RHAMM protein expression is low in lung colonies compared to primary tumors and STAT1 protein expression is also blunted (Fig. 4D) indicating impaired IFN signaling, consistent with the evidence that RHAMM expression-loss provides protection from STING-IFN induced apoptosis. These collective data identify a tumor-intrinsic, RHAMM-dependent mechanism for promoting tumor cell survival that favors metastatic lung colonization despite a DNA damage burden.

## Discussion

Our results identify a novel tumor-intrinsic mechanism resulting from *Rhamm*-loss that provides a survival advantage in the lung microenvironment and that associates with clonal dominance. We identify specific properties of the lung microenvironment (high ROS, TGFB levels), which combine with a *Rhamm*-dependent tumor-intrinsic impairment of DNA damage sensing to provide a selective survival/growth advantage in this tissue but not in the mammary gland microenvironment.

Advanced genomic and diagnostic technologies identify metastasis as an extremely complex process that can result from single or multiple clones arising early or late in the genetic evolution of primary tumors [53]. Tumor clonal genetic heterogeneity is considered to be a driving force in both successful metastatic colonization and treatment resistance. Each step in the colonization of extraneous tissues exerts selective pressure, which results in a genetic and epigenetic heterogeneity that is distinct from the primary tumor. However, the factors driving this selection process are still poorly understood. Therapeutic strategies remain largely based upon analyses of primary tumors [53], and this knowledge gap contributes to the present restricted ability to eradicate and/or manage metastases. Our results identify one mechanism, loss of RHAMM signaling, that associates with reduced genetic diversity and, paradoxically, clonal amplification of lung colonies that are resistant to ROS-mediated DNA damage.

Positive selection, detected by SNV similarities, is clearly evident in *Rhamm*^*−*/−^ primary mammary tumors even though phenotypic properties are not detectably altered. Selection is strongly amplified in lung tissue metastatic tumors, as indicated by an almost 100-fold enrichment in shared SNVs, which predicts that the lung microenvironment creates a bottleneck limiting expansion of metastatic clones. The enhanced tumor-intrinsic survival capability in the lung is linked to loss of RHAMM expression and blunting of STING/IFN signaling in both *Rhamm*^−/−^ and Wildtype genotypes. Interestingly, RHAMM expression-loss in Wildtype lung metastatic colonies is not associated with the increased SNV homogeneity observed in *Rhamm*^−/−^ lung tumor colonies, suggesting that aberrant STING-IFN survival responses alone are not responsible for clonal homogeneity. We analyzed genic SNVs for clues as to mechanisms that might complement this *Rhamm*^−/−^ intrinsic tumor cell phenotype. It is remarkable that while Wildtype lung metastases share few genic SNVs (Additional file 2: Table S3), and these mutant genes do not group onto the same signalling pathways (data not shown), *Rhamm*^−/−^ lung metastases collectively share an extraordinary 125 genic SNVs. None of these mutations associate with IFN or TGFB-regulated signalling as queried by KEGG, IPA or GSEA data sets (IPA shown, Additional file 2: Table S6), but in silico analyses predict a role for these mutations in cancer and wound repair. Wound and cancer microenvironments share many immune and stromal fingerprints, suggesting that critical changes in the microenvironment of *Rhamm*^−/−^ primary tumors may contribute to positive selection of homogeneous clones. Another consideration is evidence that RHAMM expression contributes to maintenance of embryonic stem cell pluripotency [20] and is a marker for a subpopulation of renewing tumor stem cells [54–56], which are thought to drive tumor cell heterogeneity. *Rhamm*-loss may increase clonal homogeneity, in part, by restricting pluripotency of these tumor cell subsets. Further multiplexed and spatial platform analyses are required to identify the tumor-intrinsic and host mechanisms that are regulated by RHAMM, and that underlie the clonal selection and expansion of mammary tumor cells.

Evidence is emerging that the oncogenicity of many dominant oncogenic driver genes is context- and tissue-dependent [36, 48, 49]. For example, high expression of CD44, like RHAMM, is linked to breast cancer initiation yet both proteins can perform metastasis suppressor functions in experimental models of breast cancer susceptibility [26]. Both genes are subject to alternative splicing of mRNA, and selective expression of isoforms can have different tumorigenic effects. For example, expression of one of two RHAMM isoforms is oncogenic in an experimental model of pancreatic cancer [9]. Our results probe the duality in oncogenic functions of RHAMM by providing mechanistic insight into the conditions favoring the metastasis suppressor over promoter functions using RHAMM as an example. This type of contextual and mechanistic information is critical for successful targeting of many multifunctional proteins typified by context-dependent oncogenic and suppressor properties [6, 57]. Thus, while a large body of data predict that RHAMM acts as a tumor promoter in breast cancer [13, 15], our results uncover an unexpected role for RHAMM in suppressing metastasis under the very specific conditions of whole animal *Rhamm*-loss. In addition to identifying and providing context for the dual oncogenic and tumor suppressor functions of RHAMM, our results raise the possibility of using RHAMM expression as a biomarker for sensitivity to interferon therapy, which, as an example, can impact immune therapy effectiveness in tumor management [58, 59].

In summary, our conclusions from the present study are that RHAMM contributes to primary tumor and metastasis clonal heterogeneity by regulating tumor cell survival via STING/IFNG/STAT1 signaling in microenvironments characterized by a high potential for DNA damage. When RHAMM is lost, these properties do not detectably impact primary tumor initiation or growth but favor survival and growth of homogeneous metastatic clones in lung tissue that are under positive selection as a result of host *Rhamm*-loss.

## Supplementary Information

**Additional file 1.** Supplementary materials and methods.

**Additional file 2.** Supplementary figures and tables.

## Data Availability

The datasets used and/or analyzed during the current study are available from the corresponding author on reasonable request.

## Abbreviations

CNV: Copy number variant
ddPCR: Digital droplet PCR
GO: Gene ontology
KEGG: Kyoto encyclopedia of genes and genomes
MDGA: Mouse diversity genotyping array
PCA: Principal component analysis
PCR: Polymerase chain reaction
RHAMM: Receptor for HA mediated motility
SNP: Single nucleotide polymorphism
SNV: Single nucleotide variant
