# Supplementary material for: RHAMM regulates MMTV-PyMT-induced lung metastasis by connecting STING-dependent DNA damage sensing to interferon/STAT1 pro-apoptosis signaling
Source: Breast Cancer Res. 2023 Jun 22;25:74. doi: 10.1186/s13058-023-01652-1 (PMC10286489; doi:10.1186/s13058-023-01652-1)
Supplement: Supplementary file 1 — Additional file 1. Supplementary materials and methods. [file 13058_2023_1652_MOESM1_ESM.docx]

**SUPPLEMENTARY MATERIALS AND METHODS**

**Mouse breeding, genotyping, tumor measurements and mammary gland whole mount preparation**

All animal experiments were previously approved by the animal use committee of Western University. Mice were housed in standard cages, water and food were given *ad libidum*. MMTV-PyMT male mice were obtained from the Jackson Research laboratory, CD44^-/-^ mice had been previously obtained from the Karlsruhe Research Center, Institute for Genetics (1) and *Rhamm*^-/-^ mice had been generated in the laboratory of Prof. Anton Berns at the Netherlands Cancer Institute, Amsterdam, The Netherlands (2). *Rhamm* Exons 8-16 were deleted by homologous recombination in mouse embryonic stem (ES) cells. This involved electroporation of ES cells with a DNA construct consisting of the *Hprt* selection marker flanked by homologous genomic *Rhamm* DNA. ES cells that integrated the construct into the *Rhamm* gene were identified by Southern Blots. These ES cells were injected into C57Bl/6 blastocysts, resulting in chimeric and, after breeding with FVB mice, heterozygous *Rhamm*^+/-^ mice. These heterozygous mice were backcrossed to C57Bl/6 mice for more than 10 generations before *Rhamm*^+/-^ x *Rhamm*^+/-^ breeding was used to generate homozygous *Rhamm*^-/-^ mice. Genotyping was performed as previously described (2). *CD44*^-/-^:*Rhamm*^-/-^ mice were generated by *CD44*^-/-^ x *Rhamm*^-/-^ breeding followed by brother-sister mating of the heterozygous *CD44*^+/-^:*Rhamm*^+/-^ F1 generation. We followed the breeding plan published by Lopez et al. (3) to generate *CD44*^-/-^:MMTV-PyMT, *Rhamm*^-/-^:MMTV-PyMT and *CD44*^-/-^:*Rhamm*^-/-^:MMTV-PyMT mice. Homozygous *CD44*^-/-^, *Rhamm*^-/-^ or *CD44*^-/-^:*Rhamm*^-/-^ were crossed with FVB MMTV-PyMT transgenic mice for one generation and the heterozygous F1 generation was backcrossed to the appropriate homozygous mice for one generation. Control wildtype MMTV-PyMT with a comparable FVB:C57Bl/6 background were generated by breeding C57Bl/6 mice with MMTV-PyMT mice for one generation and backcrossing the resulting F1 mice to C57Bl/6 mice for one generation. Offspring of the F1 backcrosses were used for tumor analysis. All mice were housed for 16 weeks and tumor development was followed by at least twice/week palpation and caliper measurements. At 16 weeks, mice were sacrificed by CO_2_ inhalation, primary R4 tumours and lungs were removed and either fixed in 3 % PFA or snap frozen in dry ice.

**Whole mount preparation**

Whole mounts were prepared as described (4). Briefly, mice were sacrificed by CO_2_ inhalation. Using small scissors, one skin incision was made across the abdomen and a second incision down the midline. The skin was pealed back and the mammary gland removed. The mammary gland was transferred to an electrostatically adherent microscopy slide and blunt end forceps used to spread out the gland over the glass slide. Slides were stored in Carnoy’s solution over night at RT. Slides were stained with Carmine alum solution over night at RT. Excess dye was removed by treating slides with de-staining solution at RT, until solution remained clear. Whole mounts were dehydrated by treating slides with 70, 80, 95 and 100 % ethanol for 15 min each, then slides were submerged in xylene over night at RT. Slides were mounted with Permount SP15. After drying for several days, whole mounts were imaged using a dissecting microscope.

**Buffer preparation**

Carnoy’s solution: For 1 L Carnoy’s buffer mix 750 ml 100 % Ethanol with 250 ml Glacial Acetic Acid under a chemical hood.

Carmine Alum: For 100 ml Carmine Alum add 200 mg Carmine dye and 500 mg Aluminum potassium to 100 ml demineralized H_2_O. Bring the mixture to a short boil, then let it cool down before use.

**De-staining solution**

For 1 L de-staining solution mix 700 ml 100 % Ethanol with 298 ml demineralized H_2_O and add 2 ml 12 N HCl.

**SNP genotype and CNV analysis**

A reference set of 114 classical laboratory mouse strains was used as a training set for SNP genotyping and as a germline comparison through genetic analyses in this study (5).

**Select genic CNV confirmation by droplet digital PCR (ddPCR)**

DNA quantity and quality were assessed prior to ddPCR, using a NanoDrop 2000c spectrophotometer (Thermo Fisher Scientific, Waltham, Massachusetts, USA), and diluted to approximately 8 ng/μl. Subsequently, the DNA was fragmented by centrifuging 140 μl of DNA sample at 16 000xg for 3 min in a QIAshredder column (Qiagen, Venlo, Limburg, Netherlands).

Each PCR reaction, with the exception of negative controls, contained 5 μl of DNA template, 5 μl of PCR-grade water, 12.5 μl of the ddPCR™ Supermix for Probes (Bio-Rad, Hercules, California, USA), 1.25 μl of the FAM™ dye-labelled TaqMan® assay for the gene target of interest, 1.25 μl of the VICR dye-labelled TaqMan^®^ reference assay. 20 μl of the PCR mixture was used for droplet generation and PCR. Droplets were generated by a QX200™ droplet generator (Bio-Rad, Hercules, California, USA). PCR was carried out in a C1000 Touch™ thermal cycler (Bio-Rad, Hercules, California, USA) with the following program: 1 cycle at 95°C for 10 min, 45 cycles of denaturation at 95°C for 30 s, annealing and extension at 60°C for 1 min and enzyme deactivation at 98°C for 10 min. Droplets were read using a QX200™ droplet reader and analyzed with QuantaSoft™ software (v1.7.4.0917; Bio-Rad, Hercules, California, USA).

**SNP and CNV phenogram construction**

SNP distance was calculated by totaling the number of loci where pairs of samples did not share the same genotype call and dividing by the total number of SNP loci. Loci where both mice had a No Call genotype were not counted as a difference. For pairwise CNV distance calculations, SNP and IGP markers were assigned the copy number state (0, 1, 2, or 3+) that they called as their “genotype”. The total number of CNV genotype differences between pairs of samples was divided by the total number of SNP and IGP loci to obtain CNV genetic distance values. SNP and CNV distance matrices are available in Supplementary Materials. The genetic distance matrices were used to construct a phenogram file using the BIONJ function of the APE package (v3.3) for R (v3.2.2), which implements the algorithm described by (6). The phenogram file was saved in Newick format and uploaded to Figtree (v1.4.2) to generate coloured phenogram images.

**Selection for SNP loci with functional annotation**

SNP probes at 493,290 SNP loci were annotated locally with Sequence Ontology terms using Ensembl’s Variant Effect Predictor (7). SNPs that could not be annotated using the GRCm38.p4 mouse reference assembly were excluded from further analyses. In addition, only probes with one functional annotation term were used in subsequent analyses. The Y and mitochondrial chromosomes were not analyzed given the small number of SNP loci represented on the MDGA (8). Based on these criteria, a set of 330,298 SNP loci was used for further analysis.

**Removal of SNP loci from genotype analyses given variation expected due to the mixed MMTV-PyMT genetic background**

From the set of 330 298 SNP loci associated with a functional annotation, all potential SNP loci with genotypic differences that could be attributed to the known genetic diversity in the MMTV-PyMT transgenic mice (9) were removed from further analyses. To do this, genotyping calls at 330 298 SNP loci were compared between seven C57BL/6J (3 females, 4 males), six C57BL/6NJ (3 females, 3 males), one FVB/NJ (male), and one 129S1/SvImJ (male) mouse. SNP genotyping for these mice was performed by M.E.O. Locke using the same parameters as described previously (5). All SNP loci that showed variation in genotype among these samples were removed from further analyses, given the intent to identify candidate *de novo* postzygotic mutations. A total of 220 615 SNP loci remained and were used for all subsequent analyses. These loci were considered capable of detection of *de novo* somatic mutations that arose with tumorigenesis and metastasis from the germline mouse genetic background.

**Identification of candidate *de novo* mutations at SNP loci from the MMTV-PyMT mouse background and *de novo* mutations in each tissue**

SNP genotypes of the primary mammary tumor were compared to SNP genotypes of the MMTV-PyMT mouse background at 220 615 loci. Genotypic differences between the primary mammary tumor and the MMTV-PyMT mouse background were considered candidate *de novo* mutations at SNP loci in the primary mammary tumor. Based on the known MMTV-PyMT mouse background, the direction of the change was also inferred. Loci that had a “No Call” genotype were not considered in identifying the mouse tissue genotype. Loci that showed a change in both alleles were counted as two candidate *de novo* mutations. An example of a change in both alleles would be if the MMTV-PyMT mouse background had an AA genotype and the primary mammary tumor had a BB genotype. This method was repeated for the samples of lung tissue with metastases.

Pairwise comparisons were performed between the primary mammary tumor and the lung tissue with metastases of the same mouse and for each of the mice in order to identify candidate tissue-specific *de novo* mutations. Genotypic differences between the two tissues were used to identify candidate *de novo* mutations unique to the primary mammary tumor, and candidate *de novo* mutations unique to the lung tissue with metastases. This was performed for both wildtype and *Rhamm^-/-^*mice. Genotypic differences from the MMTV-PyMT mouse background, but not between two tissues were classified as *de novo* mutations shared by both tissues.

**DNA and RNA isolation**

DNA was isolated from primary tumors and lungs using the Wizard Genomic DNA Purification kit (Promega) and following the manufacturer’s instructions for tail DNA isolation. In brief, 600 µl of EDTA/nuclei lysis solution plus 17.5 µl of 20 mg/ml Proteinase K (Roche) were added to the tissue. Samples were incubated ON at 55 °C. 3 µl RNase were added to each sample followed by a 30 min incubation at 37 °C. 200 µl of Protein Precipitation solution was added, and samples were kept on ice for 5 min. Samples were centrifuged for 4 min at 13 000 rpm at RT. The supernatant was removed and added to 600 µl isopropanol. Samples were centrifuged for 4 min at 13 000 rpm at RT. Supernatant was removed. DNA pellets were washed with 70% cold ETOH, centrifugation was repeated, and the supernatant was removed. DNA pellets were allowed to dry for at least 2 hrs at RT. DNA was re-hydrated in 100 µl Rehydration Solution, ON at 4°C. DNA concentration was measured using a NanoDrop™ instrument. RNA was isolated from primary tumors using Trizol and following the manufacturer’s instructions. Frozen tumor samples were homogenized in 2 ml Trizol buffer. Lysates were mixed with 400 µl Chloroform followed by 15 min centrifugation at 13 000 rpm, 4°C. The supernatant was removed and the RNA was precipitated with 1 ml Isopropanol at -20°C. Samples were centrifugated for 40 min at 13 000 rpm, 4°C, washed with 70% EtOH, and centrifugated again. The supernatant was removed and the pellets were air dried. Pellets were dissolved in 200 µl RNAse free water. RNA was reprecipitated by adding 1/10 Vol Na-acetate and 2.5 Vol 100 % EtOH and storing samples overnight at -20°C. Centrifugation and wash steps were repeated and RNA pellets were air dried. Pellets were dissolved in 50 µl RNASE free water and concentration was determined using a NanoDrop™ instrument.

**RNA sequencing**

All samples were sequenced at the London Regional Genomics Centre (Robarts Research Institute, London, Ontario, Canada; http://www.lrgc.ca) using the Illumina NextSeq 500 (Illumina Inc., San Diego, CA). Total RNA samples were quantified using the NanoDrop (Thermo Fisher Scientific, Waltham, MA) and quality was assessed using the Agilent 2100 Bioanalyzer (Agilent Technologies Inc., Palo Alto, CA) and the RNA 6000 Nano kit (Caliper Life Sciences, Mountain View, CA). They were then processed using the Vazyme VAHTS Total RNA-seq (H/M/R) Library Prep Kit for Illumina (Vazyme, Nanjing, China) which includes rRNA reduction. Briefly, samples were rRNA depleted, fragmented, cDNA was synthesized, indexed, purified and amplified via PCR. Libraries were then equimolar pooled into one library and size distribution was assessed on an Agilent High Sensitivity DNA Bioanalyzer chip, and quantitated using the Qubit 2.0 Fluorimeter (Thermo Fisher Scientific, Waltham, MA). The library was sequenced on an Illumina NextSeq 500 as a 76 bp single end run, using one High Output v2 kit (75 cycles). Fastq data files were analyzed using Partek Flow (St. Louis, MO).

After importation, data was aligned to the *Mus musculus* mm10 genome using STAR 2.7.3a and annotated using Ensemble v100. Features with less than 14 reads were filtered out, followed by normalization by CPM (Counts Per Million and add 0.0001). Fold change and p-values were determined using Partek Flow’s Gene Specific Analysis (GSA) between *Rhamm*^-/-^ versus wildtype groups.

**PCR array**

 2 μg total RNA was reverse transcribed using the RT^2^profiler reverse transcription kit (Qiagen) following the manufacturer’s instructions. RT^2^ Profiler DNA damage repair PCR arrays (Qiagen) were used and analyzed following manufacturer’s instructions.

**Western blot analysis**

Cells or tissue samples were lysed in RIPA buffer containing Halt Proteinase and Phosphatase inhibitor cocktail (ThermoScientific). Protein concentration was determined using the Pierce BCA protein quantification kit and following manufacturer’s instructions. Protein samples were mixed 1:4 with 4x Laemmli sample buffer (BioRad) and incubated at 95 ºC for 5 min. 30-60 ug protein were loaded onto a Bolt 4-12 % Bis-Tris Plus gel (Invitrogen). Electrophoreses was performed using mini gel tanks (Life Technologies) and 1 x Running buffer (Thermo Scientific). Proteins were transferred onto Immobilon-P transfer membranes using 1 x Transfer buffer (Thermo Scientific) containing 10 % methanol. For blocking of nonspecific binding sites, membranes were incubated with TBST containing 5 % skim milk over night at 4 ºC. Primary antibodies (STAT, Cell Signaling #9172, RHAMM, abcam ab124729, GAPDH, Invitrogen MA5-15738) were diluted 1:500 – 1: 2000 in TBST containing 1 % skim milk. Membranes were incubated with 5 ml antibody solution for 2 hrs at RT followed by two 15 min wash steps with TBST 1 % skim milk. Membranes were incubated with 1:5000 diluted anti rabbit-HRP or anti mouse-HRP secondary antibodies for 2 hrs at RT. Secondary antibodies were removed by washing membranes with TBST 1 % skim milk for 45 min. Western blots were developed using Immobilon Forte Western HRP substrate (Millipore).

**Cell migration and invasion assays**

Random cell migration: Cells were plated on fibronectin coated IBIDI 8-well chambers using a cell number that achieved a 25 % cell density after overnight culture. Cell migration was followed by video microscopy using a Nikon Eclipse TE300 microscope equipped with heated cell culture chamber (IBIDI) and digital camera. The NSI Elements AR Analysis program (Nikon) was used to track the migration path for each cell over time. Migration speed was defined as the total distance travelled/ time. Directional persistence was defined as the total distance travelled from origin divided by the net path length.

Scratch wound assay: Cells were plated on fibronectin coated IBIDI 8-well chambers using enough cells to achieve a confluent monolayer after overnight culture. Cell monolayer were scratched with a blue pipette tip and dead cells were washed off with culture medium. Cell migration was followed by video microscopy as described above. Scratch wound closure was determined by measuring the distance of the cell migration front at time 0 and 24 hrs.

Invasion assays: 200 000 cells were plated in 24 well cell culture inserts using culture medium in the upper and lower chamber. After 48 hrs, cells in the upper chamber were removed using a Q-tip. Cells that migrated through the membrane were fixed in paraformaldehyde/PBS for 10 min at RT, washed in PBS and mounted with ProLong Gold antifade reagent with DAPI. Cell nuclei were counted using an upright microscope.

**Immunohistochemistry (IHC) and Immunofluorescence (IF) of tissue sections**

In brief, tissue sections were deparaffinized and re-hydrated by xylene (2 x 10 min each), 100 %, 95 %, 70 %, EtOH, H_2_O, 1x PBS, 5min each. For antigen retrieval, sections were boiled for 20 min in 10 mM Na-Citrate pH 6 using a microwave.  Sections were allowed to cool down to RT and washed in 1 x PBS for 5 min at RT. For IF: Non -specific antibody binding was blocked by incubation in 3 % BSA/PBS for at least 45 min at RT. Primary antibodies (RHAMM, abcam #124729, gamma H2AX, abcam #26350, cleaved Caspase 3, Cell Signaling #9661, Vimentin, abcam #92547, Ki67, abcam #16667, STAT1, Cell Signaling #9172, Pan KRT, abcam #27988, ZEB1, abcam #87280) were diluted in 1 % BSA/PBS and sections were incubated ON at 4 ºC using a humidified chamber. The following day, sections were washed 3 x 5 min in 1 x PBS at RT. Secondary antibodies were diluted in 1% BSA/PBS and sections were incubated for 1 hr at RT, in a humidified chamber. After 1 hr incubation, sections were washed 3 x 5 min in 1 x PBS at RT. Sections were mounted with DAKO fluorescent mounting medium and imaged using an Olympus confocal. For IHC: Endogenous peroxidase activity was blocked by incubating sections with peroxide/PBS for 10 min at RT. Sections were washed in 1 x PBS for 5 min at RT. Blocking of non-specific antibody binding and incubation with biotinylated primary antibody was performed as described for IF staining. The next day, sections were washed with PBS 3 x 5 min at RT. For detection of primary antibodies, sections were incubated with 1:500 diluted Streptavidin-HRP for 1 hr at RT. Sections were washed 3 x 5 min in 1x PBS. Antibody detection was performed using DAB+ kit, following the manufacturer’s instructions. Sections were washed with H_2_O, counterstained with hematoxylin, mounted with Cytoseal (ThermoScientific) and imaged using an upright microscope (Olympus).

**Tumor cell isolation**

16-week-old mice were sacrificed by CO_2_ inhalation and the primary tumors were isolated. Tumor tissue was minced into small pieces using scalpels. Tissue pieces were added to 10 ml DMEM/F12 medium containing 0.2 % Collagenase A and incubated at 37 °C, 100 rpm for 45 min. The cell suspension was centrifuged at 1 300 rpm for 5 min. The supernatant was removed, and the pellet was resuspended in DMEM/F12 medium. The cell suspension was centrifuged at 1 000 rpm for 5 sec. The supernatant was removed, the pellet was resuspended in growth medium (DMEM/F12, 5 % FBS, Streptomycin, 20 nM estradiol, transferrin, insulin, 5 ng/ml mouse EGF) and plated on cell culture plates. Medium was changed twice/week until cultures were confluent. Confluent cultures were passaged once using Trypsin/EDTA or cells were frozen in freezing medium (90 % FBS, 10 % DMSO) and stored in liquid nitrogen.

Py8119 cells were obtained from the ATCC (ATCC CRL-3278) and cultured in F12K medium (Wisent) supplemented with 5 % FBS and Mito+ Serum Extender. These cells had been isolated and cloned from tumors that arose in C57Bl/6 MMTV-PyMT mice and therefore don’t contain genomic sequences from FVB mice (10).

**ROS/NOS and 8-oxodG ELISA**

ROS/NOS was detected in primary tumors and lungs using the Oxi Select *in vitro* ROS/RNS assay kit (CellBiolabs). Manufacturer’s instructions were followed. Primary tumors and lungs were homogenized in PBS at 4 ºC. A BioTek Synergy H4 reader was used for quantification. 8-oxodG was detected in primary tumors and lungs using the HT 8-oxo dG ELISA kit (TREVIGEN). Manufacturer’s instructions were followed. A BioTek Synergy H4 reader was used for quantification.

**Comet Assay**

Frozen samples of cells that had been isolated from primary tumors were shipped to Trevigen, Inc (Gaithersburg, MD). DNA strand breaks were quantified by Trevigen, Inc using an alkaline Comet assay protocol.

**Apoptag**^®^**staining of tissue sections and cells**

Apoptag^®^ staining was performed using the Apoptag^®^ Fluorescein *In Situ* Apoptosis Detection Kit (Millipore). Manufacturer’s instructions for staining of paraffin embedded sections and adherent cells were followed. In brief, paraffin embedded sections were deparaffinized by passing sections through xylene and ETOH series as described under IHC staining. Sections were treated with Proteinase K (20 μg /ml PBS) for 15 min at RT. Sections were washed with PBS, 2 x 2 min each. Sections were treated with Equilibration buffer for 10 sec at RT. After equilibration, sections were treated with working strength TdT enzyme and incubated at 37 ºC for 1 hr. Sections were washed with Wash Buffer for 10 min at RT. Sections were washed 3 x 1 min with PBS. Sections were covered with Anti-digoxigenin conjugate and incubated 30 min at RT. Sections were washed four times with PBS at RT. Sections were mounted with ProLong Gold antifade reagent with DAPI (Thermo Fisher Scientific).

Adherent cells were fixed in 4 % paraformaldehyde/PBS pH 7.4 for 10 min at RT. Cells were washed 2 x 5 min in PBS. Cells were then post-fixed in cold Ethanol:Acetic Acid 2:1 for 5 min at -20 ºC. Cells were washed 2 x 5 min in PBS. Equilibration buffer was applied for at least 10 sec. at RT. Working strength TdT enzyme was applied and cells were incubated for 1 hr at 37ºC. Cells were incubated for 10 min in wash buffer at RT. Cells were washed three times in PBS. Anti-digoxigenin conjugate was applied and cells were incubated for 30 min at RT. Cells were washed four times in PBS and mounted with ProLong Gold antifade reagent with DAPI (Thermo Fisher Scientific). Samples were imaged using an Olympus confocal. Staining was quantified for the green and blue channel using Image J and the ratio green/blue pixels was calculated.  Alternatively, total number of cells and cells with green nuclei were counted and the percentage of positive (green) nuclei calculated.

**SUPPLEMENTARY REFERENCES**

1. Schmits R, Filmus J, Gerwin N, Senaldi G, Kiefer F, Kundig T, et al. CD44 regulates hematopoietic progenitor distribution, granuloma formation, and tumorigenicity. Blood. 1997;90(6):2217-33.

2. Tolg C, Poon R, Fodde R, Turley EA, Alman BA. Genetic deletion of receptor for hyaluronan-mediated motility (Rhamm) attenuates the formation of aggressive fibromatosis (desmoid tumor). Oncogene. 2003;22(44):6873-82.

3. Lopez JI, Camenisch TD, Stevens MV, Sands BJ, McDonald J, Schroeder JA. CD44 attenuates metastatic invasion during breast cancer progression. Cancer Res. 2005;65(15):6755-63.

4. Tolg C, Yuan H, Flynn SM, Basu K, Ma J, Tse KCK, et al. Hyaluronan modulates growth factor induced mammary gland branching in a size dependent manner. Matrix Biol. 2017;63:117-32.

5. Locke ME, Milojevic M, Eitutis ST, Patel N, Wishart AE, Daley M, et al. Genomic copy number variation in Mus musculus. BMC Genomics. 2015;16:497.

6. Gascuel O. BIONJ: an improved version of the NJ algorithm based on a simple model of sequence data. Mol Biol Evol. 1997;14(7):685-95.

7. McLaren W, Gil L, Hunt SE, Riat HS, Ritchie GR, Thormann A, et al. The Ensembl Variant Effect Predictor. Genome Biol. 2016;17(1):122.

8. Yang S, Wang L, Huang J, Zhang X, Yuan Y, Chen JQ, et al. Parent-progeny sequencing indicates higher mutation rates in heterozygotes. Nature. 2015;523(7561):463-7.

9. Guy CT, Cardiff RD, Muller WJ. Induction of mammary tumors by expression of polyomavirus middle T oncogene: a transgenic mouse model for metastatic disease. Mol Cell Biol. 1992;12(3):954-61.

10. Gibby K, You WK, Kadoya K, Helgadottir H, Young LJ, Ellies LG, et al. Early vascular deficits are correlated with delayed mammary tumorigenesis in the MMTV-PyMT transgenic mouse following genetic ablation of the NG2 proteoglycan. Breast Cancer Res. 2012;14(2):R67.
