# Supplementary material for: RHAMM regulates MMTV-PyMT-induced lung metastasis by connecting STING-dependent DNA damage sensing to interferon/STAT1 pro-apoptosis signaling
Source: Breast Cancer Res. 2023 Jun 22;25:74. doi: 10.1186/s13058-023-01652-1 (PMC10286489; doi:10.1186/s13058-023-01652-1)

**SUPPLEMENTARY FIGURES**

**
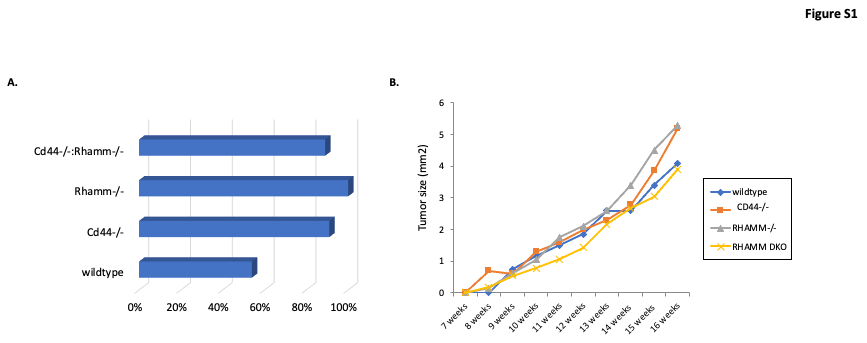
**

**Figure S1. Loss of Rhamm, CD44 or both Rhamm and CD44 increases MMTV-PyMT dependent lung metastasis but has no effect of primary tumor growth.**

A. H&E stained lung tissue sections of 16 weeks old mice were used to score the presence of lung metastases/mouse. Graph shows percentage of mice with lung metastasis. B. Primary tumor growth was followed over time by palpation of R4 mammary glands. Graph shows tumor size increase over time.

**
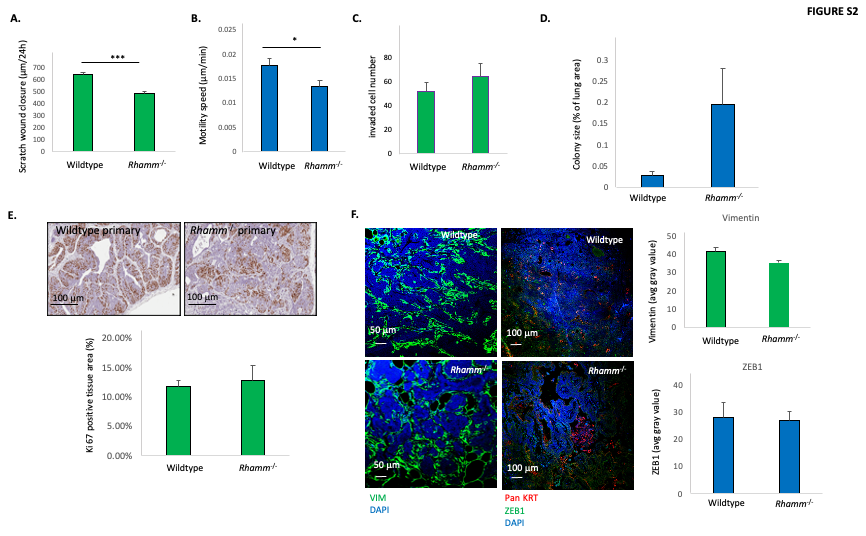
**

**Figure S2.** ***Rhamm*-loss reduces tumor cell motility but does not alter invasion, mesenchymal plasticity of growth**. A. Scratch wounds were placed on confluent cultures of Wildtype and *Rhamm*^-/-^ mammary tumor cell monolayers as described in Methods. The re-covering of scratch wounds was followed in real-time using time-lapse cinemicrography and quantified as the area of wound closure over time as described in Methods. *Rhamm^-/-^* mammary tumor cells recover significantly less of the scratch wound than Wildtype tumor cells. Values are the Mean and S.E.M. *n* = 3 biological replicates ****p* < 0.001. B. Mammary tumor cell motility speed of leading cells responding to scratch wounding was quantified using Nikon Elements software as described in Methods. *Rhamm*^-/-^ tumor cells migrate significantly more slowly than Wildtype comparators. Values are the Mean and S.E.M. *n* = 30 tumour cells/genotype. **p* < 0.05. C. Invasion of Wildtype and *Rhamm*^-/-^ tumor cells were analyzed in Boyden chamber assays using filter with 8 μM pores. Graphs show number of invaded cells, which is similar for both genotypes. Values are the Mean and S.E.M of *n* = 25 fields/genotype. D. H&E stained lung sections were used to quantify size of Wildtype and *Rhamm*^-/-^ metastatic lung colonies. Nodules were outlined and area was quantified as described in Methods. Graph depicts colony size measured as % tissue area/colony. Significant differences in colony size of Wildtype vs. *Rhamm*^-/-^ lung tumors are not detected. Values are the Mean and S.E.M. n=20 colonies/genotype. E. Primary tumor cell proliferation was quantified by Ki67 staining of histology sections as described in Methods. Brown staining: Ki67 positive nuclei, blue staining: hematoxylin counter staining. *Rhamm*-loss has no detectable effect on tumor cell proliferation. Values are the Mean and S.E.M. n=5 tumors/genotype. E. Wildtype and *Rhamm*^-/-^ primary tumors were stained for the EMT markers VIMENTIN, ZEB1 and pan cytokeratin. Confocal images were used for quantification as described in Methods. Differences in expression of these genes are not detected. Values are the Mean and S.E.M of *n* = 3 samples/genotype

**
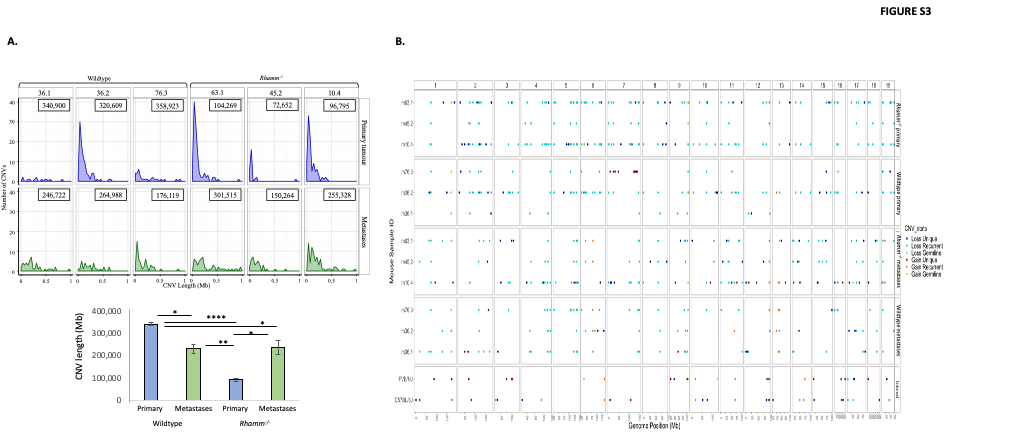
**

**Figure S3. Wildtype and *Rhamm*^-/-^ CNV’s are similar in number and genome distribution but differ in length.** A. Distribution of CNV events across the autosomes for Wildtype and *Rhamm^-/-^* primary tumors and lung metastases compared with two inbred mouse stocks C57BL/6J (*n* = 8) and FVB/NJ (*n* = 1) used as the reference for germline (inherited) CNV burden. Different colours represent the state and recurrence of CNV events, specifically unique loss (blue), recurrent loss (turquoise), germline loss (green), unique gain (red), recurrent gain (orange), and germline gain (yellow) defined as described in Methods. The CNVs of both Wildtype and *Rhamm*^-/-^ tumors occur across the genome characteristic of a mutator phenotype. B. Distribution of CNV lengths for primary tumor and lung metastases from three Wildtype and three *Rhamm^-/-^* mice. Primary tumor CNV lengths are coloured in blue and those of metastases are coloured in green. Individual mice are represented by the numeric identifiers at the top of the figure. Inset numbers are average CNV length (bp) for each genotype and tumor type. Wildtype (WT) primary tumor CNVs are significantly longer than lung metastases and CNVs of *Rhamm^-/-^* (Rh) tumors. In contrast, CNV’s of *Rhamm*^-/-^ primary tumors are significantly shorter than *Rhamm*^-/-^ lung metastases and Wildtype tumors. *p<0.05, **p<0.01. ****p<0.0001


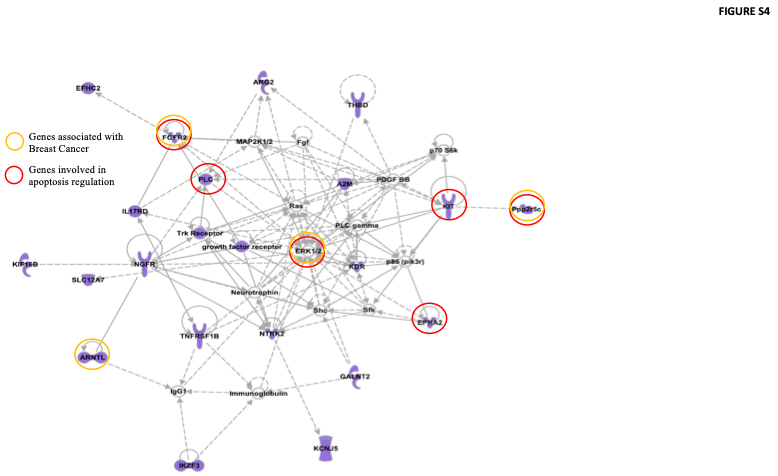


**Figure S4. Mutations detected as changes in genotypes at SNP loci observed in *Rhamm^-/-^* metastases implicate multiple genes involved in apoptosis regulation** Top IPA network in which molecules encoded by genes containing *de novo* mutations at SNP loci shared by all three samples of metastases in *Rhamm^-/-^* mice. Proteins in purple represent proteins encoded by genes containing *de novo* mutations at SNP loci with non-neutral functional annotations.


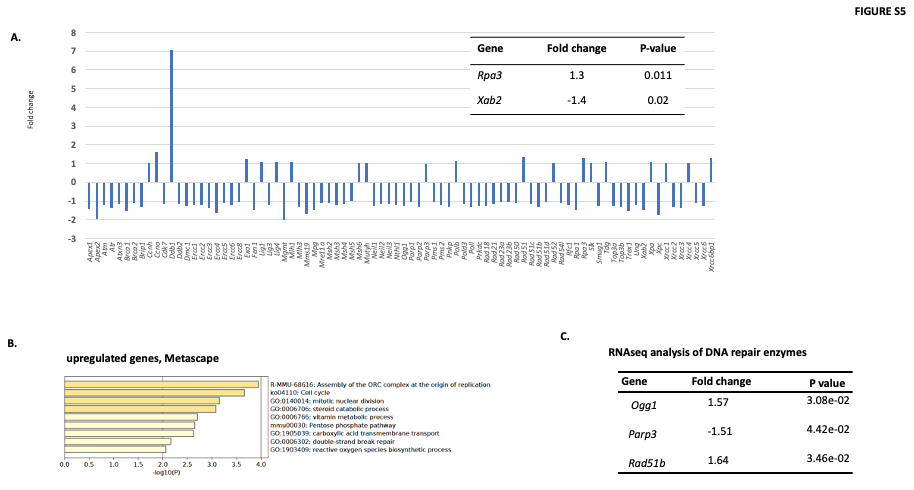


**Figure S5. Rhamm-loss does not strongly affect expression of DNA damage repair genes.** A. PCR array of DNA repair genes was performed as described in Methods and reveals very minor changes in the expression of two DNA repair enzymes. Values are shown as fold changes based upon 3 replicates. Statistical significance was detected using a two-way ANOVA. B, C. RNA-seq and transcriptome was performed as described in Methods. B. Metascape analysis of genes whose mRNA expression is up-regulated by *Rhamm*-loss associate with cell cycle changes. C. Analysis of RNA-seq data reveals few changes in the mRNA expression of DNA repair enzymes.

SUPPLEMENTARY TABLES

**Table S6. Functional enrichment analysis using Ingenuity Pathway Analysis (IPA) for the shared *de novo*mutations at SNV loci observed in *Rhamm^-/-^*tissues**


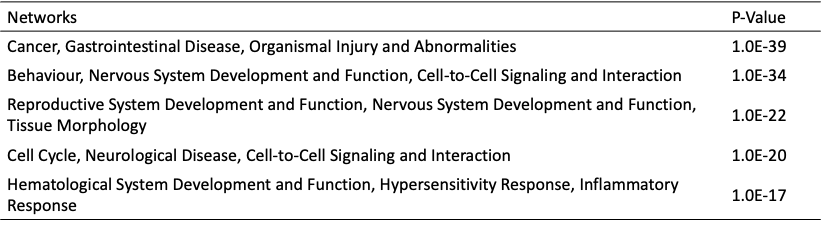

Supplement: Supplementary file 2 — Additional file 2. Supplementary figures and tables. [file 13058_2023_1652_MOESM2_ESM.docx]
